# Whole-Exome Sequencing Reveals a Rare Variant of *OTOF* Gene Causing Congenital Non-syndromic Hearing Loss Among Large Muslim Families Favoring Consanguinity

**DOI:** 10.3389/fgene.2021.641925

**Published:** 2021-05-25

**Authors:** Mohd Fareed, Varun Sharma, Inderpal Singh, Sayeed Ur Rehman, Gurdarshan Singh, Mohammad Afzal

**Affiliations:** ^1^PK-PD Formulation and Toxicology Division, CSIR Indian Institute of Integrative Medicine, Jammu, India; ^2^Academy of Scientific and Innovative Research (AcSIR), Ghaziabad, India; ^3^Ancient DNA Laboratory, Birbal Sahni Institute of Palaeosciences, Lucknow, Uttar Pradesh, India; ^4^BioinfoRes, Jammu, India; ^5^Department of Biochemistry, School of Chemical and Life Sciences, Jamia Hamdard, New Delhi, India; ^6^Human Genetics and Toxicology Laboratory, Section of Genetics, Department of Zoology, Aligarh Muslim University, Aligarh, India

**Keywords:** hearing disorders, hearing impairment, deaf mutes, autosomal recessive (AR) diseases, cousin marriages, Indian population, Jammu and Kashmir (J and K), genetic counseling

## Abstract

Non-syndromic hearing loss (NSHL) is one of the most frequent auditory deficits in humans characterized by high clinical and genetic heterogeneity. Very few studies have reported the relationship between *OTOF* (Locus: DFNB9) and hereditary hearing loss in India. We aimed to decipher the genetic cause of prelingual NSHL in a large affected Muslim consanguineous families using whole-exome sequencing (WES). The study was performed following the guidelines and regulations of the Indian Council of Medical Research (ICMR), New Delhi. The population was identified from Jammu and Kashmir, the Northernmost part of India. Near about 100 individuals were born deaf-mute in the village of 3,000 inhabitants. A total of 103 individuals (with 52 cases and 51 controls) agreed to participate in this study. Our study revealed a rare non-sense homozygous mutation NC_000002.11:g.2:26702224G>A; NM_001287489.2:c.2122C>T; NP_001274418.1:p.(Arg708^∗^) in the 18th exon of the *OTOF* gene. Our study provides the first insight into this homozygous condition, which has not been previously reported in ExAC, 1,000 Genome and genomAD databases. Furthermore, the variant was confirmed in the population cohort (*n* = 103) using Sanger sequencing. In addition to the pathogenic *OTOF* variant, the WES data also revealed novel and recurrent mutations in *CDH23, GJB2, MYO15A, OTOG*, and *SLC26A4* genes. The rare pathogenic and the novel variants observed in this study have been submitted to the ClinVar database and are publicly available online with the accessions SCV001448680.1, SCV001448682.1 and SCV001448681.1. We conclude that *OTOF*-related NSHL hearing loss is prevalent in the region due to successive inbreeding in its generations. We recommend premarital genetic testing and genetic counseling strategies to minimize and control the disease risk in future generations.

## Introduction

Over 446 million people have disabling hearing loss worldwide. This estimate is projected to accelerate over 630 million by 2,030 and may raise up to 900 million in 2,050 (Olusanya et al., 2019). The global prevalence of prelingual hearing loss is 1 in 500 newborns and is the 4th leading cause of disability among living individuals (Duman and Tekin, 2012). Phenotypically, hereditary hearing loss (HHL) can be classified into syndromic hearing loss (SHL) and non-syndromic hearing loss (NSHL). The NSHL is one of the most frequent sensory deficits in humans characterized by high clinical and genetic heterogeneity. Approximately 90% of NSHL cases from severe to profound congenital deafness exhibit an autosomal-recessive (AR) pattern of inheritance (DFNB forms). The prevalence of prelingual NSHL is approximately 2.7 cases per 1,000 live births (Vona et al., 2015).

Genotype-phenotype correlations help in understanding the mechanistic etiology, progression, and prognosis of the inherited genetic disorder. However, in case of NSHL, the diagnosis becomes more challenging due to high clinical complexity and genetic heterogeneity. The molecular elucidation of such complex disorder with precise genomic approaches can provide genetic origin and functional consequences, which could be better implemented for proper medical investigation, prognostic, and therapeutic targets. To date, a total of 116 NSHL genes have been identified^1^. Out of these, 45 genes for autosomal dominant-NSHL (AD-NSHL), 5 genes for X-linked and about 75 genes are linked with AR-NSHL. The product of these genes has multiple roles in maintaining the normal physiology of the inner ear. Mutation in any of these genes may have a deleterious impact, altering the normal hearing physiology (Shearer et al., 1993).

Hearing loss is a severe disorder but grossly neglected in India. According to World Health Organization (2018) data, the prevalence of auditory deficit in the country is over 63 million (about 6.3%) of the total population. The population of India and other South Asian countries provides much complexity due to the admixture of their genomes during evolutionary timescales (Patterson et al., 2012). To discover genetic variations of the heterogenetic disorder in such an ethnic group and/or geographically isolated population may need highly equipped and rigorous diagnostic approaches. Massive parallel sequencing (MPS) or next-generation sequencing (NGS) technologies provide an opportunity to precisely explore the genetic architecture of the disease, which furthermore could be used as a baseline for medical genetic testing (Shearer et al., 2010). Very few studies have reported the relationship between *OTOF* (Locus: DFNB9) and HHL in India.

Here, we employed the whole-exome sequencing (WES) approach to precisely identify the functional pathogenic mutations causing congenital hearing loss from the Muslim population favoring consanguinity.

## Materials and Methods

### Study Population

**FIGURE 1:**
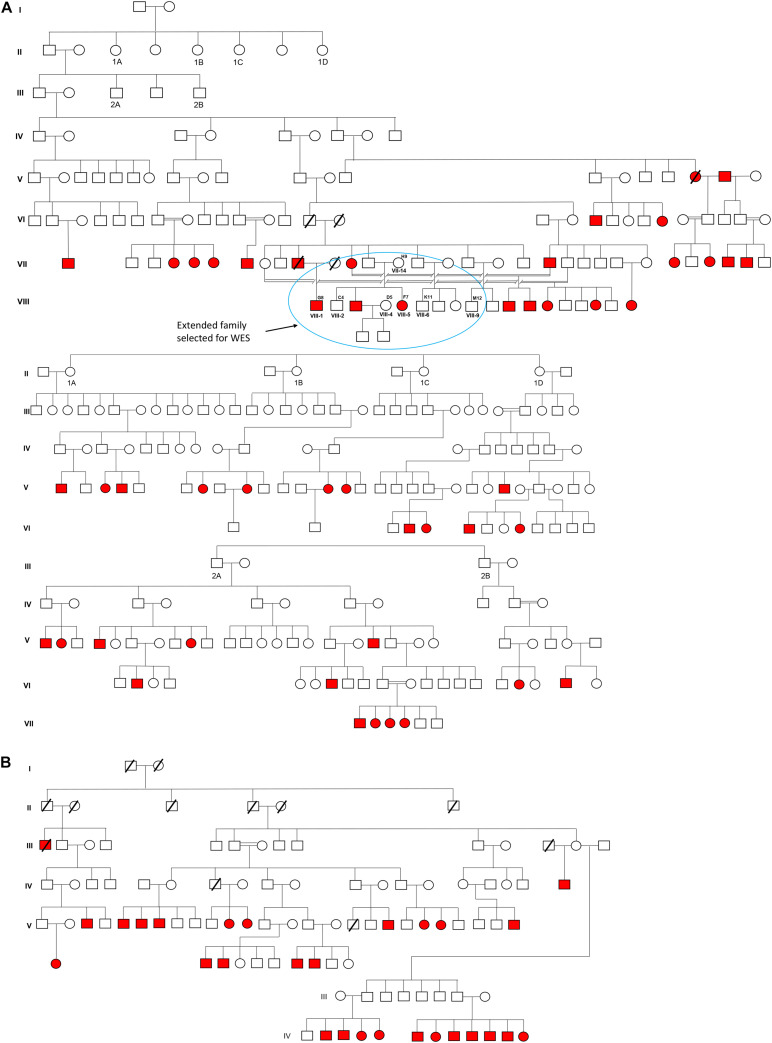
Pedigree and inheritance of deaf-mute syndrome in the families. **(A,B)** are the two large family trees and **(A)** have two sub-family trees expatiated from its first and second generations. The extended family from the first family tree (circled blue) was considered for whole-exome sequencing. The squares and circles represent males and females, respectively, where the cases are presented in red color.

The cohort was selected from the Doda District of Jammu and Kashmir, North India. Approximate 3,000 residents inhabited this village, belonging to the Muslim community favoring consanguinity in their ancestry. To date, in this village, more than 100 individuals were born deaf-mute; some of them migrated to other states (i.e., Punjab, India) and a few to nearby districts. In our preliminary survey, we identified 74 deaf-mute live and 3 deceased cases. Figure 1 presents the pedigree and inheritance detail of all 77 cases. Out of 74 deaf-mutes, only 52 cases were agreed to participate in the study. A total of 103 individuals aged 20–60 years (with 52 cases and 51 controls) were recruited for this study. WES was performed in 07 samples with 02 cases (i.e., F7 and G8) and 05 controls (i.e., C4, D5, H9, K11, and M12), while the rest (*n* = 96) were subjected to Sanger sequencing to confirm the candidate pathogenic variant.

### Sample Collection, DNA Isolation and Quantification

Blood samples were collected using a 21-gage syringe to draw up to a volume of 5 ml in a vacutainer containing Tris-EDTA. For saliva samples, the participants were provided with collection tubes and were asked to provide a 2.5 ml sample. The participants were instructed to spit into the tube up to the red fill line marked on the tube (approximately 2.5 ml), excluding the froth. DNA was extracted using QIAamp DNA Mini Kit 50 (Cat# 51304, Qiagen). The DNA samples were was subjected to QIAXPERT for quantifying the amount of DNA and the purity was checked by measuring the 260/280 nm ratio. DNA samples were also subjected to agarose gel electrophoresis and, after passing through DNA quality check (Supplementary Table 1), were proceeded for Library Protocol.

### Whole-Exome Sequencing and Bioinformatic Analysis

The WES library was prepared using Agilent-Sure Select XT Reagent Kit, Illumina (ILM) platforms. Biotinylated oligonucleotide capture probes (V5 + UTR), also called baits, that was designed for the human exons was provided with the kit and used to enrich the region of interest (whole exome) by hybridization. The workflow involved shearing of DNA, repairing ends, adenylation of 3’ ends, followed by adapter ligation. At each step, the products were purified using AMPure beads. The adapter sequence was added onto the ends of DNA fragments to generate paired-end libraries. The resulting adaptor-ligated library was purified, quantified and hybridized with an exome-specific biotinylated capture library. After hybridization, the targeted molecules were captured on streptavidin beads. The resulting enriched DNA library was multiplexed by adding index tags by amplification, followed by purification. Indexed captured library DNA was assessed to check the quality and quantity of the captured libraries. The sequencing was carried out in Illumina HiSeq X10 to generate 2X–150 bp sequence reads at an average 100− sequencing depth. Only those samples were considered for data processing that surpasses the quality scores (Q30 value) greater than 75% of the sequenced bases. The base quality distribution, base content and the GC content are presented in Supplementary Figure 1. The overall alignment percentage (alignment to GRCh37/hg19) in all the samples was greater than 97% (Supplementary Table 2). The average coverage and on-target percentage for the samples was around 99 and 90%, respectively (Supplementary Table 3). The distribution of sequencing depth is shown in Supplementary Figure 2. The sequencing reads were processed and analyzed using the BROAD Institute’s Genome Analysis Toolkit (GATK-Toolkit) (DePristo et al., 2011) and variant calling was performed using the complete human reference genome (hg19, NCBI release GRCh37).

The bioinformatic pipelines (alignment variant calling and variant annotation) used in our study are shown in Supplementary Figure 3. All common polymorphisms with a minor allele frequency (MAF) higher than 0.01 were filtered out using several public databases such as 1,000 genomes database (1000 Genomes Project Consortium et al., 2015), Ensembl GRCh37 genome browser (Zerbino et al., 2018), exome aggregation consortium database (ExAC) (Lek et al., 2016), genome aggregation database (gnomAD) (Karczewski et al., 2020), and database of single nucleotide polymorphisms (dbSNP). The ClinVar database was used to check the previously reported mutations and associated phenotypes. Exclusion of intronic, synonymous, inframe insertions/deletions (InDels) and mutations in untranslated regions whereas the missense, non-sense variations and frameshift InDels located in exons or splice sites were prioritized. The remaining variants were then verified in dbSNP and NCBI databases.

### *In silico* Evaluation for the Pathogenicity of Candidate Mutation

The altered amino acid was checked for its evolutionary conservation across different species, including the primates and mammals using the genome browser of the University of California at Santa Cruz (UCSC) (Kent et al., 2002). *In silico* programs including MutationTaster2 (Schwarz et al., 2014), PolyPhen-2 (Adzhubei et al., 2010) and scale-invariant feature transform (SIFT) (Kumar et al., 2009) were used to predict the possible impact of the detected variants.

### Audiometric Characteristics

The pulse tone audiometric (PTA) records of the subjects were noted. The hearing level grades were categorized according to WHO and the National Hearing Test (NHT) guidelines viz., normal (<20 dB), mild (20–40 dB), moderate (41–70 dB), severe (71–90 dB), and profound (>90 dB). The average values of both the ears (left and right) were considered to calculate the hearing threshold levels. The age of the individual was also recorded at the time of audiometry.

### Co-segregation Analysis

Whole-exome sequencing (at 100X depth) provides sufficient details for the confirmation of the candidate variant. However, Sanger sequencing has also been performed to re-validate/or reconfirm the mutation identified by targeted NGS. Primers (PXL-A0145439) for exon 18 of the *OTOF* gene (reference sequence NM_001287489.2 and Chr2:26669916-26791779 context region in hg19), were manufactured and supplied by Pxlence^2^. The thermal cycler program was set according to the manufacturer’s instructions, using the conditions: initial incubation 98°C for 2 min, 98°C for 20 sec, followed by 35 cycles at 60°C for 30 s, 72°C for 40 s, final extension 72°C for 10 min and hold at 4°C. PCR products were confirmed using a 1.7% w/v agarose gel electrophoresis. The PCR products were then processed for cycle sequencing/BigDye terminator assay, followed by Sephadex (column-based) purification. Finally, the PCR products were loaded onto DNA Sequencer (SeqStudio Genetic Analyzer).

## Results

### Identification of a Pathogenic Mutation Using Whole-Exome Sequencing

**FIGURE 2:**
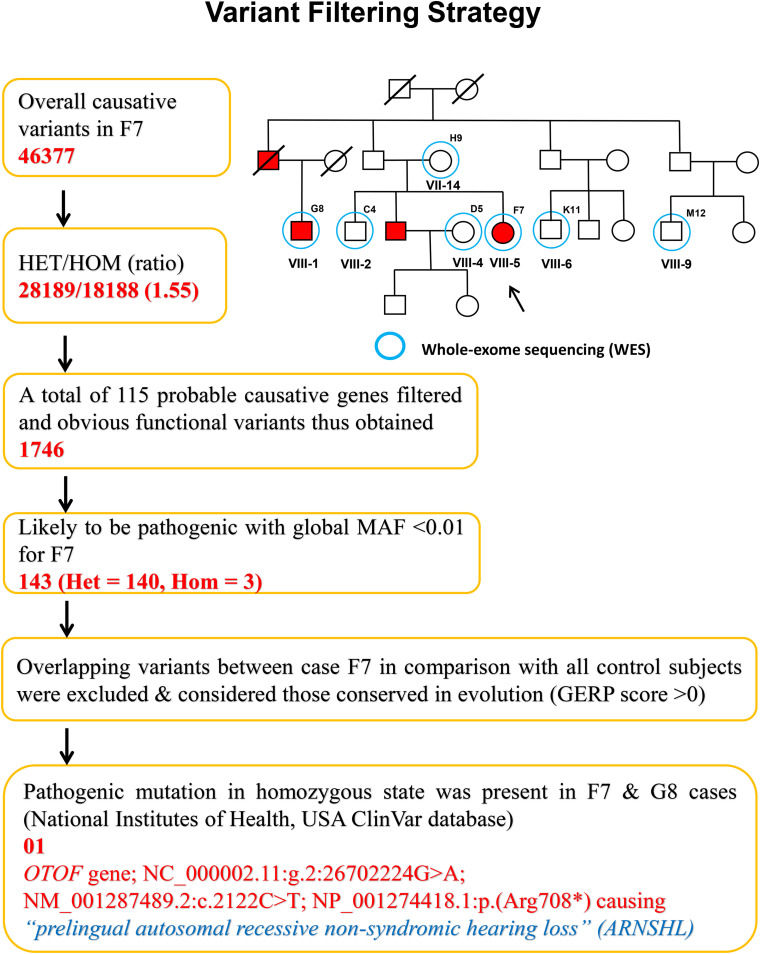
Variant filtering strategy. The narrow down approach applied to whole exome-sequenced samples to explore the most promising causative mutation.

The variants produced from exome-sequencing are presented in Supplementary Tables 4–6. WES analysis generated approximately 72,000 genetic variants (including SNPs, insertions and deletions) in each sample. By applying the narrow down filtering approach, the number of probable causative mutations are presented in Supplementary Tables 7–9, respectively. The variant filtering strategy used to find out the most promising causative mutation has been presented in Figure 2. The most promising causative variants have been presented in Supplementary Table 10. After the removal of duplicates and common mutations, the variants were filtered for rare (<1%), evolutionary conserved and functional homozygous recessive mutations using the online GenIO database (an integrated pipeline based on RefGene, NHLBI-ESP, 1,000 Genomes, dbSNP, ClinVar, COSMIC, gnomAD, OMIM and M-CAP databases) following the American College of Medical Genetics and Genomics and the Association of Molecular Pathology (ACMG-AMP) guidelines (Koile et al., 2018). We observed approximately 03 homozygous and 140 heterozygous mutations (likely to be pathogenic, global MAF <0.01) among the two cases (F7 and G8). After the removal of overlapping variants with controls and considering the conserved in evolution (GERP score >0), we thus identified the disease-causing rare variant NC_000002.11:g.2:26702224G > A; NM_001287489.2:c.2122C > T; NP_001274418.1:p.(Arg708^∗^) (rs80356590) in 18th exon of the *OTOF* gene. This *OTOF* variant is very rare and no homozygous variant previously reported in genomAD, ExAC, and 1,000 Genome databases; however, few heterozygous cases have been reported in genomAD. We confirmed the *OTOF* mutation using BAM and VCF files in IGV 2.5.3 software.

The functional consequence (*in silico* validation) of the variant was predicted using Mutation Taster (Table 1), which revealed the deleterious consequence homozygous condition by premature terminating the otoferlin protein at 708 amino acid. The substitution of the arginine by a premature stop codon p.(Arg708^∗^) produces a truncated variant of otoferlin remains non-functional in the sensory hair cells, potentially causing profound hearing loss.

**TABLE 1:** Functional evidence of the *OTOF* variant (NC_000002.12:g.2:26702224G > A) by *in silico* evaluation, using Mutation Taster.

Start (aa)	End (aa)	Feature	Details	Impact
1	1963	TOPO_DOM	Cytoplasmic	Lost
792	821	COILED		Lost
947	1052	DOMAIN	C2 3	Lost
1088	1088	CONFLICT	L = P (in Ref. 1; AAD26117 and 2; AAG12992/AAG17468)	Lost
1303	1310	COMPBIAS	Poly-Lys	Lost
1314	1320	COMPBIAS	Poly-Glu	Lost
1479	1577	DOMAIN	C2 4	Lost
1787	1787	CONFLICT	G = S (in Ref. 5; BAG58982)	Lost
1964	1984	TRANSMEM	Helical	Lost
1965	1983	COMPBIAS	Poly-Leu	Lost
1985	1997	TOPO_DOM	Extracellular	Lost

The stop codon insertion by the variant (NM_001287489.2:c.2122C > T) terminates the protein at 708 position (NP_001274418.1:p.Arg708*), causing the loss of various functional domains.

In addition to the rare pathogenic *OTOF* variant, the WES data also revealed some novel and rare recurrent genomic mutations in the extended family. The novel variants (not found in ExAC, 1,000 G and genomAD databases) of *OTOG* NM_001292063.2:c.5438T > G; NP_001264198.1: p.(Val1813Gly) and *SLC26A4* NM_000441.2:c.1668T > A NP_000432.1:p.(Tyr556^∗^) and the recurrent variants of *CDH23* NM_022124.6:c.4892C > T NP_071407.4:p.(Ala1631Val), *GJB2* NM_004004.6:c.493C > T NP_003995.2:p.(Arg165Trp), *MYO15A* NM_016239.4:c.5894G > A NP_057323.3:p. (Arg1965His) and others (presented in Supplementary Table 10) were observed in heterozygous/or compound heterozygous conditions. The candidate pathogenic (*OTOF*) and the novel variants (*OTOG* and *SLC26A4*) have been submitted to ClinVar database and are publicly available online with the accessions SCV001448680.1, SCV001448682.1, and SCV001448681.1, respectively.

### Audiometric and Genotypic Characteristics

**FIGURE 3:**
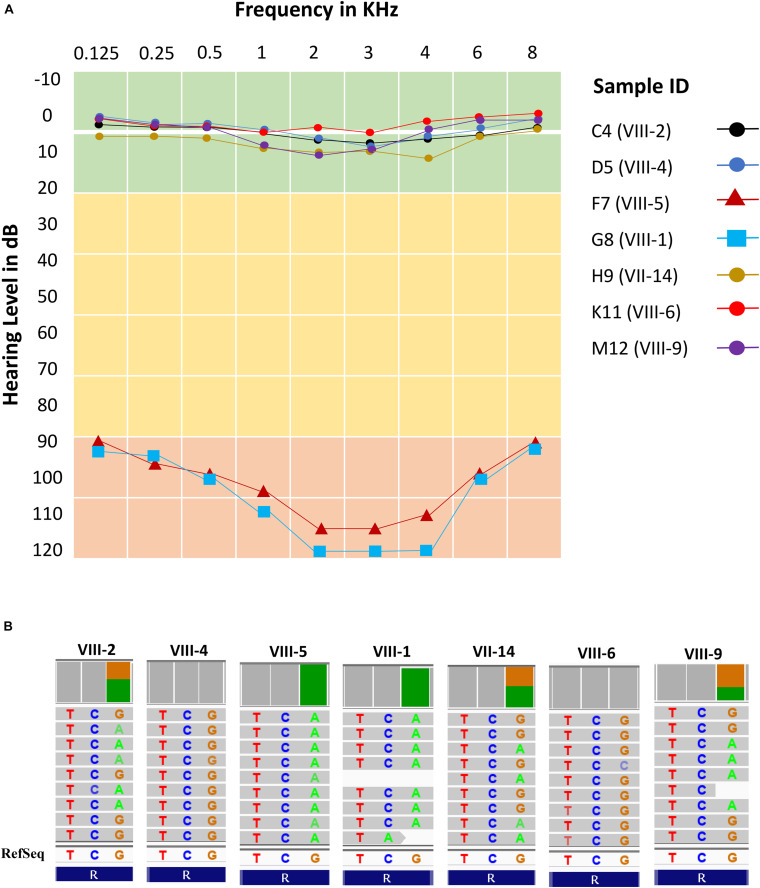
Audiometric characteristics and whole exome data. **(A)** Audiometric data shows five normal hearing subjects (levels between 0 and 10 dB) and two cases (levels >90 dB) for all frequencies (KHz). **(B)** Whole exome data reflects exactly the phenotypic data with two homozygous cases (F7 and G8), three heterozygous normal (C4, H9, and M12) and two normal with homozygous reference allele (D5 and K11).

All subjects (cases and controls) showed normal clinical features (i.e., no structural and observable phenotypic deformity) other than hearing. The audiometric and genomic characteristics of proband have been presented in Figure 3. The normal individuals (control) showed the hearing level in between 0–20 dB at frequencies 0.125, 0.25, 0.5, 1, 2, 3, 4, 6, and 8 kHz. However, the threshold level of deaf-mute subjects falls under a profound (>90 dB) category for the same audio frequencies (Figure 3A). The cases F7 (aged 31 years), G8 (aged 25 years) exhibit the characteristics of profound prelingual NSHL. The WES data of cases (F7 and G8) and controls C4 (aged 43 years), D5 (aged 35 years), H9 (aged 60 years), K11 (aged 22 years), and M12 (aged 34 years) have been shown in Figure 3B. The cases (F7 and G8) carry a homozygous mutation; among controls, the C4, H9, and M12 possess heterozygous and D5 and K11 are homozygous for the reference allele (carrying no *OTOF* mutation).

### Co-segregation Analysis

**FIGURE 4:**
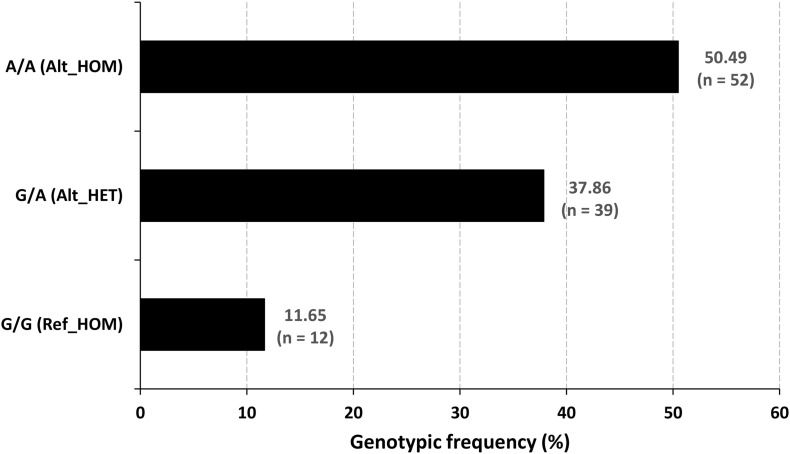
Genotypic frequency of the *OTOF* variant. The mutated homozygotes were the (*n* = 52) cases, and the normal hearing people were the reference homozygotes (*n* = 12) and heterozygous carrier (*n* = 39) genotypes.

The family pedigree and *OTOF* segregation with the disease phenotype has been well demonstrated in Supplementary Figure 4a. The next generation WES (Figure 3B) and Sanger sequencing data (Supplementary Figure 4B) provides the evidence for perfect segregation of the *OTOF* variant with the auditory phenotype. The data clearly depicts the autosomal recessive non-syndromic hearing loss (ARNSHL). Further, Sanger sequencing was performed in the remaining 96 samples to reconfirm the mutation pattern of the *OTOF* gene. The genotypic details of the candidate variant of *OTOF* gene for all 103 subjects have been displayed in Figure 4. The ancestral lineage of the subjects has a history of consanguineous marriages which determines the existence of the phenotypic spectrum of *OTOF* in the current generation.

## Discussion

The results of our study indicate that a non-sense mutation in the *OTOF* gene was terminating the peptide chain and generating a truncated protein variant, which ultimately leads to prelingual neurosensory non-syndromic DFNB9 (OMIM, #601071) hearing loss. The functional evidence of the *OTOF* relationship with DFNB9 impairment has been well established by earlier studies (Roux et al., 2006; Pangršič et al., 2012). The *OTOF*-related NSHL has been previously reported in the populations from India, Pakistan, China, Japan, Altai Republic, Korea, Spain, Turkey, and Iran (Yasunaga et al., 2000; Tekin et al., 2005; Rodríguez-Ballesteros et al., 2008; Choi et al., 2009; Wang et al., 2010; Mahdieh et al., 2012; Ñhurbanov et al., 2016; Kim et al., 2018; Iwasa et al., 2019). In addition to OTOF-related deafness, mutations in the autosomal genes like *CDH23, Claudin14, GJB2, GJB6, MYO6, MYO15A, SLC26A4, TMC1, TMIE, TMPRSS3, TRIOBP, USHIC*, and others are predominant to cause HHL among Indian and Pakistani populations (Yan et al., 2015). It is evident from the previous studies that the mutation spectrum of *GJB2, GJB6, SLC26A4*, and *TMC1* genes was much common in the ethnic groups of eastern and southern parts of India (Padma et al., 2009; Ganapathy et al., 2014; Adhikary et al., 2015; Singh et al., 2018). In the present study, we have also identified the missense/non-sense heterozygous variants in the *OTOG* p.(Val1813Gly), *SLC26A4* p.(Tyr556^∗^) *CDH23* p.(Ala1631Val), *GJB2* p.(Arg165Trp), and *MYO15A* p.(Arg1965His) genes which are predominant to cause deafness among Indian populations.

**FIGURE 5:**
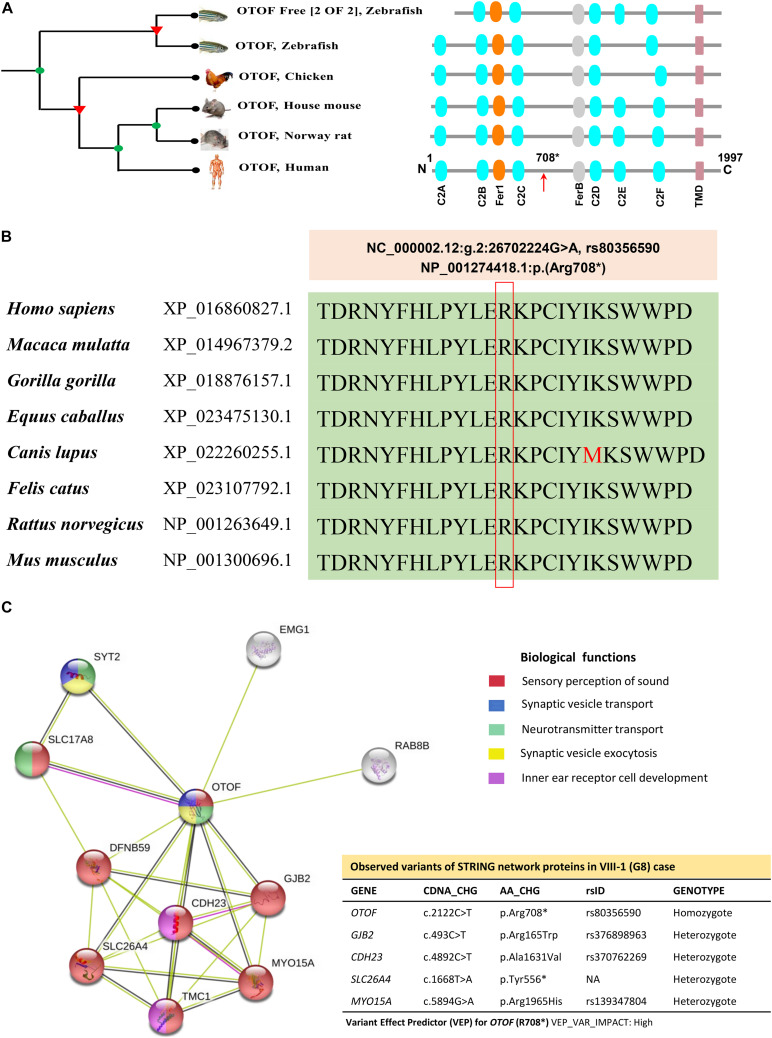
The phylogenetic relationship, conservation and association networks of OTOF protein. **(A)** The human OTOF protein domains show high resemblance with mouse and rat except for the chicken and zebrafish, which lacks the C2E domain. Ferlin1, FerlinB and Trans-membrane domains are common among all species. **(B)** The comparison of OTOF for 8 vertebrates shows a highly conserved region (at Arg708 residue) spanning between C2C and FerB domains across different vertebrates. **(C)** Network showing the functional association of *OTOF* using STRING v11.0 (https://stringdb.org/). The proximity of the proteins via threads predicts the association and their role in sensorineural activities. In the present study, exome-data have also identified the additional genomic variants in *CDH23, GJB2, MYO15A, OTOG*, and *SLC26A4* for the current STRING model.

Otoferlin is a transmembrane vesicular protein (1997 amino acids in humans) encoded by the *OTOF* gene, spanning in the short arm of chromosome 2 (2p23.3). The otoferlin is expressed in the inner hair cells (IHCs) (Liu et al., 2014) and plays a significant role in neuronal synapse and exocytosis (Shin, 2014). The protein consists of six C2 domains (C2A-F), two Ferlin conserved motifs (Fer-1 and Fer-B) and a transmembrane domain (TMD). Using the Treefam database, Schreiber et al. (2014) the close relationship of otoferlin across different species depicts the domains and motifs that were highly conserved (Figure 5A). The functional evidence for the high level of protein conservation at the site of *OTOF* mutation (NP_001274418.1:p. Arg708^∗^) in 8 vertebrates has been displayed in Figure 5B and Supplementary Figure 5a (using UCSC Genome Browser). The mRNA levels in 20 different tissues reveal the *OTOF* in the brain is highly expressed (Supplementary Figure 5b). The protein-protein interactions of OTOF using STRING v11.0 confirm the functional involvement in the neuronal synapse, exocytosis and hearing functions (Figure 5C; Szklarczyk et al., 2019).

**FIGURE 6:**
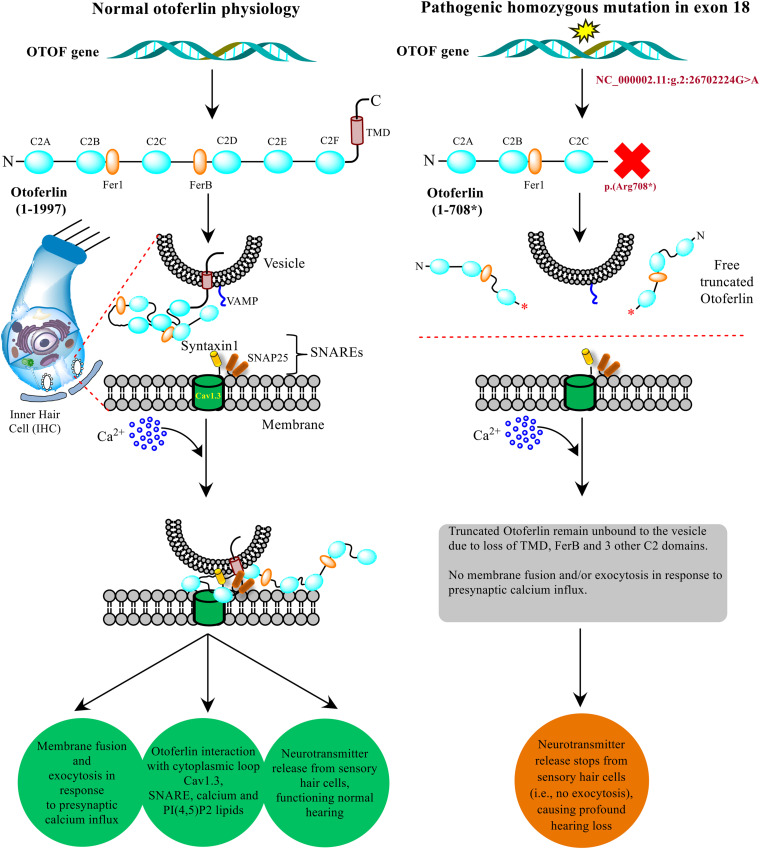
Model presenting the role of OTOF in hearing physiology. The otoferlin expressed in the inner hair cells (IHCs) cochlea typically perform the neuronal synapse and exocytosis. A non-sense mutation brings stop codon (at p. 708*) in between C2C and C2D domains results in a truncated version of the protein, which disrupts the normal function and hearing physiology. The current model was adapted from the previously well-established experimental-based studies (Ramakrishnan et al., 2014; Shin, 2014; Chatterjee et al., 2015; Hams et al., 2017).

The non-sense mutations in the *OTOF* gene producing truncated versions of protein causing DFNB9 deafness has been well-established by the researchers using *in vitro* and *in vivo* models in their experiments (Pangršič et al., 2012; Chatterjee et al., 2015; Hams et al., 2017). In the present study, a similar non-sense mutation (NM_001287489.2:c.2122C > T) results in the protein truncation at p.(Arg708^∗^) eventually altering the normal otoferlin physiology. The truncated otoferlin (N-terminus C2A-C) lacking C2D-F and TMD remain unbound with no membrane fusion or exocytosis, which ultimately halts the neurotransmitter release, causing profound hearing loss. Based upon the substantial evidence from *in silico* testing (Table 1) and previously well-established proofs of protein truncation due to different *OTOF* non-sense mutations (Chatterjee et al., 2015; Hams et al., 2017), we propose a schematic model showing the role of otoferlin protein with normal and altered physiology via p.(Arg708^∗^) in the current study (Figure 6). The otoferlin held on synaptic vesicles via TMD and C2 domains interact with the presynaptic membrane via SNAREs to execute exocytosis under Ca^2+^ influx. It is evident from the previous studies that missense mutations in C2B and C2C domains have been linked to hearing loss (Mirghomizadeh et al., 2002; Helfmann et al., 2011). N-terminal domains’ role provides structural stability to the protein and C-terminal domains (C2D-F) may play a functionally conserved or redundant role in otoferlin physiology (Chatterjee et al., 2015). Our findings predict the otoferlin synthesis stops at 708 amino acid (between C2C and FerB domains), leading to almost half protein (N-terminal with C2A-C domains) lacking the most conserved and functional part (C2D-F with TMD at C-terminus).

Parental consanguinity has been associated with an increased risk of autosomal recessive disorders (Fareed and Afzal, 2017). The consanguineous marriages with the adverse effects have been previously reported from the Muslim populations of Jammu and Kashmir (Fareed and Afzal, 2014a, b, 2016; Fareed et al., 2017). In the present study, the prevalence of prelingual hearing loss was 1 in 30, considerably higher than the global prevalence (Duman and Tekin, 2012), providing the evident consequence of inbreeding in the population. It might be possible that these groups have settled to the northernmost region of India during the evolutionary time frames and unknowingly have undergone marriages within the groups, which ultimately increased the prevalence of the disorder.

## Conclusion

The present study evaluated the ARNSHL in a tribal family of the Jammu and Kashmir region. The clinical audiometric evaluation and the information gathered from village representatives suggest the profound prelingual NSHL prevailing in the area. WES categorizes the rare pathogenic mutation in the *OTOF* gene p.(Arg708^∗^), which has been well segregated with the disease. *OTOF* plays a significant role in the neuronal synapse in the IHCs of the cochlea. The pathogenic mutation (NC_000002.11:g.2:26702224G > A) is predicted to result in the lack of the most conserved and functional domains.

The global allele frequency of this *OTOF* variant (rs80356590) is 0.00004284 (source: genomAD), which affirms the extremely rare criteria. However, in the present study population, this globally recognized rare variant of *OTOF* turns into a common one because consanguineous marriages were much prevalent in the region, ultimately increasing the risk of such autosomal recessive disorder. In summary, our study provides the roadmap and a piece of safeguard advice for clinicians and health care providers to make people familiar with the genetic cause and its increased risk in the context of inbreeding. Identification of such mutations supports better management of such disorders through genetic counseling. Establishing genetic testing facilities will help the appropriate diagnosis and opening new gateways for precision medicine. Familial and premarital screening will help in controlling the hearing disability in future generations. Further population-wide screening is needed to explore the *OTOF* penetrance and other possible genetic hideouts in NSHL among North Indian populations.

## Data Availability Statement

The rare pathogenic and the novel variants observed in this study have been submitted to the ClinVar database and are publicly available online with the accessions SCV001448680.1, SCV001448682.1, and SCV001448681.1

## Ethics Statement

The studies involving human participants were reviewed and approved by Institutional Ethical Committee (IEC), Jawaharlal Nehru Medical College (JNMC), Aligarh Muslim University, India. The patients/participants provided their written informed consent to participate in this study. All methods were performed following the guidelines and regulations of the Indian Council of Medical Research (ICMR), New Delhi.

## Author Contributions

MF, VS, IS, SUR, GS, and MA conceived and designed the study. MF analyzed and interpreted the data. MF, VS, SUR, GS, and MA contributed to the drafting and critical review of the manuscript. All authors contributed to the article and approved the submitted version.

## Conflict of Interest

IS was employed by company BioinfoRes, Jammu, India. The remaining authors declare that the research was conducted in the absence of any commercial or financial relationships that could be construed as a potential conflict of interest.

## Footnotes

1 http://hereditaryhearingloss.org/

2 https://www.pxlence.com/index.php/

## Web Resources

GenIO, http://bioinformatics.ibioba-mpsp-conicet.gov.ar/GenIO/index.php/

gnomAD, https://gnomad.broadinstitute.org/

Hereditary Hearing Loss, http://hereditaryhearingloss.org/

Mutation Taster, http://www.mutationtaster.org/, https://www.mutationdistiller.org/

NCBI ClinVar, https://www.ncbi.nlm.nih.gov/clinvar/

NCBI dbSNP, https://www.ncbi.nlm.nih.gov/snp/

OMIM, https://www.omim.org/

PolyPhen-2 http://genetics.bwh.harvard.edu/pph2/

SIFT, http://sift.jcvi.org/

STRING, https://stringdb.org/

The Human Gene Mutation Database, http://www.hgmd.cf.ac.uk/ac/index.php/

TreeFam, http://www.treefam.org/family/TF316871

University of California Santa Cruz Genomics Institute Genome Browser, https://genome.ucsc.edu/.

## References

[B1] 1000 Genomes Project Consortium, Auton A. Brooks L. D. Durbin R. M. Garrison E. P. Kang H. M. (2015). A global reference for human genetic variation. *Nature* 526 68–74. 10.1038/nature15393 26432245 PMC4750478

[B2] Adhikary B. Ghosh S. Paul S. Bankura B. Pattanayak A. K. Biswas S. (2015). Spectrum and frequency of GJB2, GJB6 and SLC26A4 gene mutations among nonsyndromic hearing loss patients in eastern part of India. *Gene* 573 239–245. 10.1016/j.gene.2015.07.050 26188157

[B3] Adzhubei I. A. Schmidt S. Peshkin L. Ramensky V. E. Gerasimova A. Bork P. (2010). A method and server for predicting damaging missense mutations. *Nat. Methods* 7 248–249. 10.1038/nmeth0410-248 20354512 PMC2855889

[B4] Chatterjee P. Padmanarayana M. Abdullah N. Holman C. L. LaDu J. Tanguay R. L. (2015). Otoferlin deficiency in zebrafish results in defects in balance and hearing: rescue of the balance and hearing phenotype with full-length and truncated forms of mouse otoferlin. *Mol. Cell. Biol.* 35 1043–1054. 10.1128/mcb.01439-14 25582200 PMC4333087

[B5] Choi B. Y. Ahmed Z. M. Riazuddin S. Bhinder M. A. Shahzad M. Husnain T. (2009). Identities and frequencies of mutations of the otoferlin gene (OTOF) causing DFNB9 deafness in Pakistan. *Clin. Genet.* 75 237–243. 10.1111/j.1399-0004.2008.01128.x 19250381 PMC3461579

[B6] DePristo M. A. Banks E. Poplin R. Garimella K. V. Maguire J. R. Hartl C. (2011). A framework for variation discovery and genotyping using next-generation DNA sequencing data. *Nat. Genet.* 43 491–498. 10.1038/ng.806 21478889 PMC3083463

[B7] Duman D. Tekin M. (2012). Autosomal recessive nonsyndromic deafness genes: a review. *Front. Biosci.* 17:2213–2236. 10.2741/4046 22652773 PMC3683827

[B8] Fareed M. Afzal M. (2014a). Estimating the inbreeding depression on cognitive behavior: a population based study of child cohort. *PLoS One* 9:e109585. 10.1371/journal.pone.0109585 25313490 PMC4196914

[B9] Fareed M. Afzal M. (2014b). Evidence of inbreeding depression on height, weight, and body mass index: a population-based child cohort study. *Am. J. Hum. Biol.* 26 784–795. 10.1002/ajhb.22599 25130378

[B10] Fareed M. Afzal M. (2016). Increased cardiovascular risks associated with familial inbreeding: a population-based study of adolescent cohort. *Ann. Epidemiol.* 26 283–292. 10.1016/j.annepidem.2016.03.001 27084548

[B11] Fareed M. Afzal M. (2017). Genetics of consanguinity and inbreeding in health and disease. *Ann. Hum. Biol.* 44 99–107. 10.1080/03014460.2016.1265148 27892699

[B12] Fareed M. Kaisar Ahmad M. Azeem Anwar M. Afzal M. (2017). Impact of consanguineous marriages and degrees of inbreeding on fertility, child mortality, secondary sex ratio, selection intensity, and genetic load: a cross-sectional study from Northern India. *Pediatr. Res.* 81 18–26. 10.1038/pr.2016.177 27632780

[B13] Ganapathy A. Pandey N. Srisailapathy C. R. S. Jalvi R. Malhotra V. Venkatappa M. (2014). Non-syndromic hearing impairment in India: high allelic heterogeneity among mutations in TMPRSS3, TMC1, USHIC, CDH23 and TMIE. *PLoS One* 9:e84773. 10.1371/journal.pone.0084773 24416283 PMC3885616

[B14] Hams N. Padmanarayana M. Qiu W. Johnson C. P. (2017). Otoferlin is a multivalent calcium-sensitive scaffold linking SNAREs and calcium channels. *Proc. Natl. Acad. Sci. U.S.A.* 114 8023–8028. 10.1073/pnas.1703240114 28696301 PMC5544299

[B15] Helfmann S. Neumann P. Tittmann K. Moser T. Ficner R. Reisinger E. (2011). The crystal structure of the C2A domain of otoferlin reveals an unconventional top loop region. *J. Mol. Biol.* 406 479–490. 10.1016/j.jmb.2010.12.031 21216247

[B16] Iwasa Y.-I. Nishio S.-Y. Sugaya A. Kataoka Y. Kanda Y. Taniguchi M. (2019). OTOF mutation analysis with massively parallel DNA sequencing in 2,265 Japanese sensorineural hearing loss patients. *PLoS One* 14:e0215932. 10.1371/journal.pone.0215932 31095577 PMC6522017

[B17] Karczewski K. J. Francioli L. C. Tiao G. Cummings B. B. Alföldi J. Wang Q. (2020). The mutational constraint spectrum quantified from variation in 141,456 humans. *Nature* 581 434–443. 10.1038/s41586-020-2308-7 32461654 PMC7334197

[B18] Kent W. J. Sugnet C. W. Furey T. S. Roskin K. M. Pringle T. H. Zahler A. M. (2002). The human genome browser at UCSC. *Genome Res.* 12 996–1006. 10.1101/gr.229102 12045153 PMC186604

[B19] Kim B. J. Jang J. H. Han J. H. Park H.-R. Oh D. Y. Lee S. (2018). Mutational and phenotypic spectrum of OTOF-related auditory neuropathy in Koreans: eliciting reciprocal interaction between bench and clinics. *J. Trans. Med.* 16:330. 10.1186/s12967-018-1708-z 30482216 PMC6260760

[B20] Koile D. Cordoba M. de Sousa Serro M. Kauffman M. A. Yankilevich P. (2018). GenIO: a phenotype-genotype analysis web server for clinical genomics of rare diseases. *BMC Bioinformatics* 19:25. 10.1186/s12859-018-2027-3 29374474 PMC5787240

[B21] Kumar P. Henikoff S. Ng P. C. (2009). Predicting the effects of coding non-synonymous variants on protein function using the SIFT algorithm. *Nat. Protoc.* 4 1073–1081. 10.1038/nprot.2009.86 19561590

[B22] Lek M. Karczewski K. J. Minikel E. V. Samocha K. E. Banks E. Fennell T. (2016). Analysis of protein-coding genetic variation in 60,706 humans. *Nature* 536 285–291. 10.1038/nature19057 27535533 PMC5018207

[B23] Liu H. Pecka J. L. Zhang Q. Soukup G. A. Beisel K. W. He D. Z. Z. (2014). Characterization of transcriptomes of cochlear inner and outer hair cells. *J. Neurosci.* 34 11085–11095. 10.1523/JNEUROSCI.1690-14.2014 25122905 PMC4131018

[B24] Mahdieh N. Shirkavand A. Rabbani B. Tekin M. Akbari B. Akbari M. T. (2012). Screening of OTOF mutations in Iran: a novel mutation and review. *Int. J. Pediatr. Otorhinolaryngol.* 76 1610–1615. 10.1016/j.ijporl.2012.07.030 22906306

[B25] Mirghomizadeh F. Pfister M. Apaydin F. Petit C. Kupka S. Pusch C. M. (2002). Substitutions in the conserved C2C domain of otoferlin cause DFNB9, a form of nonsyndromic autosomal recessive deafness. *Neurobiol. Dis.* 10 157–164. 10.1006/nbdi.2002.0488 12127154

[B26] Olusanya B. O. Davis A. C. Hoffman H. J. (2019). Hearing loss: rising prevalence and impact. *Bull. World Health Organ.* 97 646–646A. 10.2471/BLT.19.224683 31656325 PMC6796666

[B27] Padma G. Ramchander P. V. Nandur U. V. Padma T. (2009). GJB2 and GJB6 gene mutations found in Indian probands with congenital hearing impairment. *J. Genet.* 88 267–272. 10.1007/s12041-009-0039-5 20086291

[B28] Pangršič T. Reisinger E. Moser T. (2012). Otoferlin: a multi-C2 domain protein essential for hearing. *Trends Neurosci.* 35 671–680. 10.1016/j.tins.2012.08.002 22959777

[B29] Patterson N. Moorjani P. Luo Y. Mallick S. Rohland N. Zhan Y. (2012). Ancient admixture in human history. *Genetics* 192 1065–1093. 10.1534/genetics.112.145037 22960212 PMC3522152

[B30] Ramakrishnan N. A. Drescher M. J. Morley B. J. Kelley P. M. Drescher D. G. (2014). Calcium regulates molecular interactions of otoferlin with soluble NSF attachment protein receptor (SNARE) proteins required for hair cell exocytosis. *J. Biol. Chem.* 289 8750–8766. 10.1074/jbc.M113.480533 24478316 PMC3979417

[B31] Rodríguez-Ballesteros M. Reynoso R. Olarte M. Villamar M. Morera C. Santarelli R. (2008). A multicenter study on the prevalence and spectrum of mutations in the otoferlin gene (OTOF) in subjects with nonsyndromic hearing impairment and auditory neuropathy. *Hum. Mut.* 29 823–831. 10.1002/humu.20708 18381613

[B32] Roux I. Safieddine S. Nouvian R. Grati M. Simmler M.-C. Bahloul A. (2006). Otoferlin, defective in a human deafness form, is essential for exocytosis at the auditory ribbon synapse. *Cell* 127 277–289. 10.1016/j.cell.2006.08.040 17055430

[B33] Schreiber F. Patricio M. Muffato M. Pignatelli M. Bateman A. (2014). TreeFam v9: a new website, more species and orthology-on-the-fly. *Nucleic Acids Res.* 42 D922–D925. 10.1093/nar/gkt1055 24194607 PMC3965059

[B34] Schwarz J. M. Cooper D. N. Schuelke M. Seelow D. (2014). MutationTaster2: mutation prediction for the deep-sequencing age. *Nat. Methods* 11 361–362. 10.1038/nmeth.2890 24681721

[B35] Shearer A. E. DeLuca A. P. Hildebrand M. S. Taylor K. R. Gurrola J. Scherer S. (2010). Comprehensive genetic testing for hereditary hearing loss using massively parallel sequencing. *Proc. Natl. Acad. Sci. U.S.A.* 107 21104–21109. 10.1073/pnas.1012989107 21078986 PMC3000272

[B36] Shearer A. E. Hildebrand M. S. Smith R. J. (1993). *Hereditary Hearing Loss and Deafness Overview.* Seattle: University of Washington.

[B37] Shin O.-H. (2014). Exocytosis and synaptic vesicle function. *Compr. Physiol.* 4 149–175. 10.1002/cphy.c130021 24692137

[B38] Singh P. K. Sharma S. Ghosh M. Shastri S. S. Gupta N. Kabra M. (2018). Spectrum of GJB2 gene variants in Indian children with non-syndromic hearing loss. *Ind. J. Med. Res.* 147 615–618. 10.4103/ijmr.IJMR_76_16 PMC6118150 30168495

[B39] Szklarczyk D. Gable A. L. Lyon D. Junge A. Wyder S. Huerta-Cepas J. (2019). STRING v11: protein-protein association networks with increased coverage, supporting functional discovery in genome-wide experimental datasets. *Nucleic Acids Res.* 47 D607–D613. 10.1093/nar/gky1131 30476243 PMC6323986

[B40] Tekin M. Akcayoz D. Incesulu A. (2005). A novel missense mutation in a C2 domain of OTOF results in autosomal recessive auditory neuropathy. *Am. J. Med. Genet. A* 138 6–10. 10.1002/ajmg.a.30907 16097006

[B41] Vona B. Nanda I. Hofrichter M. A. H. Shehata-Dieler W. Haaf T. (2015). Non-syndromic hearing loss gene identification: a brief history and glimpse into the future. *Mol. Cell. probes* 29 260–270. 10.1016/j.mcp.2015.03.008 25845345

[B42] Wang D.-Y. Wang Y.-C. Weil D. Zhao Y.-L. Rao S.-Q. Zong L. (2010). Screening mutations of OTOF gene in Chinese patients with auditory neuropathy, including a familial case of temperature-sensitive auditory neuropathy. *BMC Med. Genet.* 11:79. 10.1186/1471-2350-11-79 20504331 PMC2901213

[B43] World Health Organization (2018). *WHO Programme for Ear and Hearing Care.* 1–14. Available online at: https://www.hearingreview.com/wp-content/uploads/2019/02/WHO-2018-Activity-Report.pdf (accessed March 10, 2021).

[B44] Yan D. Kannan-Sundhari A. Vishwanath S. Qing J. Mittal R. Kameswaran M. (2015). The genetic basis of nonsyndromic hearing loss in Indian and Pakistani populations. *Genet. Test. Biomarkers* 19 512–527. 10.1089/gtmb.2015.0023 26186295 PMC4575533

[B45] Yasunaga S. Grati M. Chardenoux S. Smith T. N. Friedman T. B. Lalwani A. K. (2000). OTOF encodes multiple long and short isoforms: genetic evidence that the long ones underlie recessive deafness DFNB9. *Am. J. Hum. Genet.* 67 591–600. 10.1086/303049 10903124 PMC1287519

[B46] Zerbino D. R. Achuthan P. Akanni W. Amode M. R. Barrell D. Bhai J. (2018). Ensembl 2018. *Nucleic Acids Res.* 46 D754–D761. 10.1093/nar/gkx1098 29155950 PMC5753206

[B47] Ñhurbanov A. Y. Karafet T. M. Morozov I. V. Mikhalskaia V. Y. Zytsar M. V. Bondar A. A. (2016). Whole exome sequencing reveals homozygous mutations in RAI1, OTOF, and SLC26A4 genes associated with nonsyndromic hearing loss in altaian families (South Siberia). *PLoS One* 11:e0153841. 10.1371/journal.pone.0153841 27082237 PMC4833413

