# Supplementary material for: Whole-Exome Sequencing Reveals a Rare Variant of OTOF Gene Causing Congenital Non-syndromic Hearing Loss Among Large Muslim Families Favoring Consanguinity
Source: Front Genet. 2021 May 25;12:641925. doi: 10.3389/fgene.2021.641925 (PMC8185570; doi:10.3389/fgene.2021.641925)
Supplement: Supplementary file 1 [file Data_Sheet_1.pdf]

Supplementary material

Whole-exome Sequencing Reveals a Rare Variant of *OTOF* Gene Causing Congenital Non-syndromic Hearing Loss Among Large Muslim Families Favoring Consanguinity

Mohd Fareed[1,2*], Varun Sharma[3], Inderpal Singh[4], Sayeed Ur Rehman[5], Gurdarshan Singh[1,2], Mohammad Afzal[6]

[1]PK-PD Formulation & Toxicology Division, CSIR Indian Institute of Integrative Medicine, Canal Road, Jammu, India

[2]Academy of Scientific & Innovative Research (AcSIR), Ghaziabad, Uttar Pradesh, India

[3]Ancient DNA Laboratory, Birbal Sahni Institute of Palaeosciences, Lucknow, Uttar Pradesh, India

[4]BioinfoRes, R. S. Pura, Jammu, India

[5]Department of Biochemistry, School of Chemical and Life Sciences, Jamia Hamdard, New Delhi, India

[6]Human Genetics & Toxicology Laboratory, Section of Genetics, Department of Zoology, Aligarh Muslim University, Aligarh, Uttar Pradesh, India

*Corresponding author (M. Fareed) Tel.: +91-9622025886

Email address: m.fareed@iiim.res.in; mohdfareedk@gmail.com; fareedgenetics2012@gmail.com

Supplemental Appendix 1

Whole exome sequencing analysis report : raw data, bioinformatics and interpretation.

Table S1. DNA quantification using QIAXPERT and Qubit and purity check using 260/280nm ratio.

SAMPLE ID	Sample name	QIAXPERT (ng/µL)	A260/280 RATIO	QUBIT (ng/µL) HS	COMMENTS (QC PASS/FAIL)
229830	K11	77.8	1.13	19.1	PASS
229824	C4	1088.4	1.79	676	PASS
229831	M12	215.8	1.76	158	PASS
229829	H9	1701.8	1.83	494	PASS
229825	D5	30.7	1.44	11.2	PASS
229827	F7	97.5	1.82	90.6	PASS
229828	G8	122	1.89	51	PASS

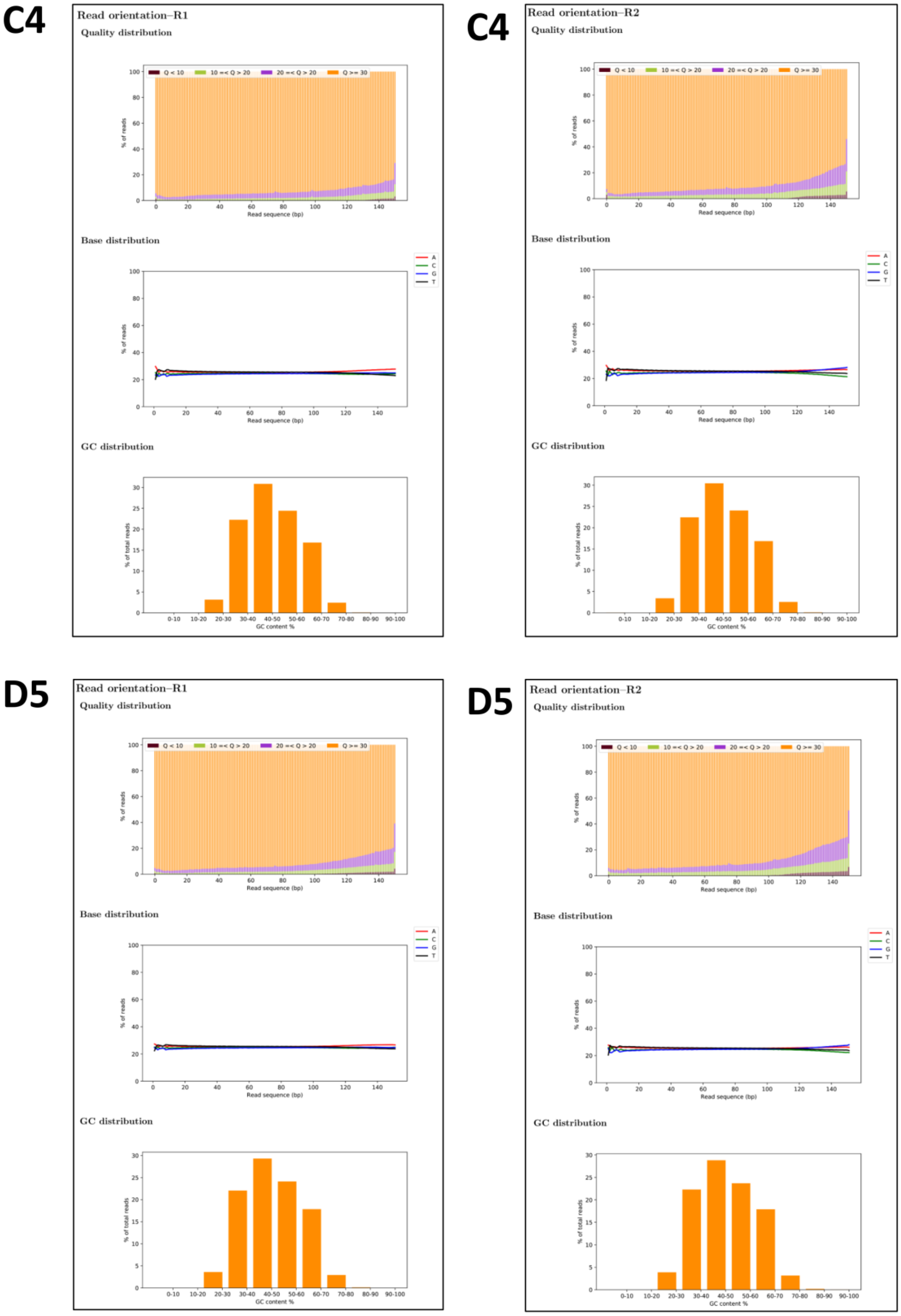

Figure S1. Quality check of reads for whole-exome sequencing (WES) strategy.

F7

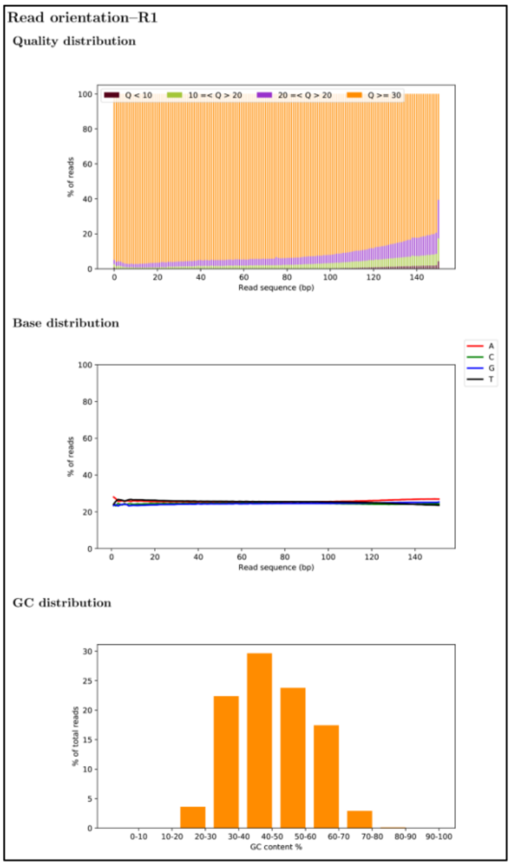

F7

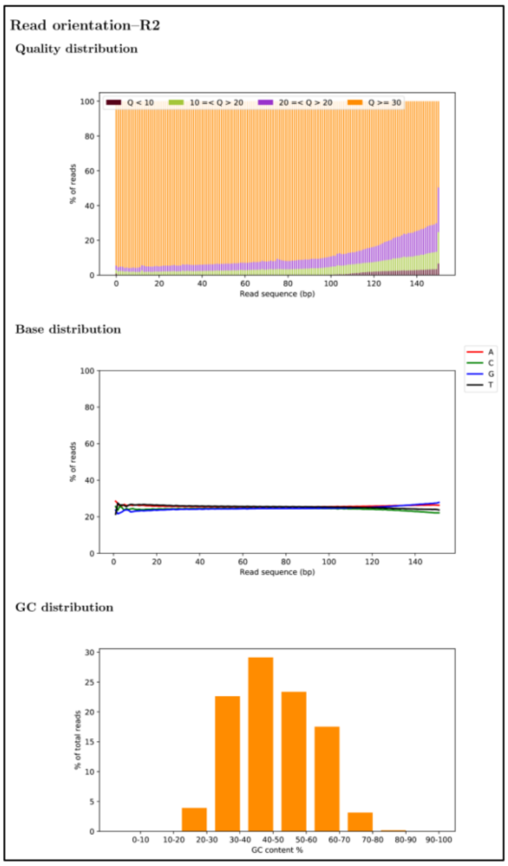

G8

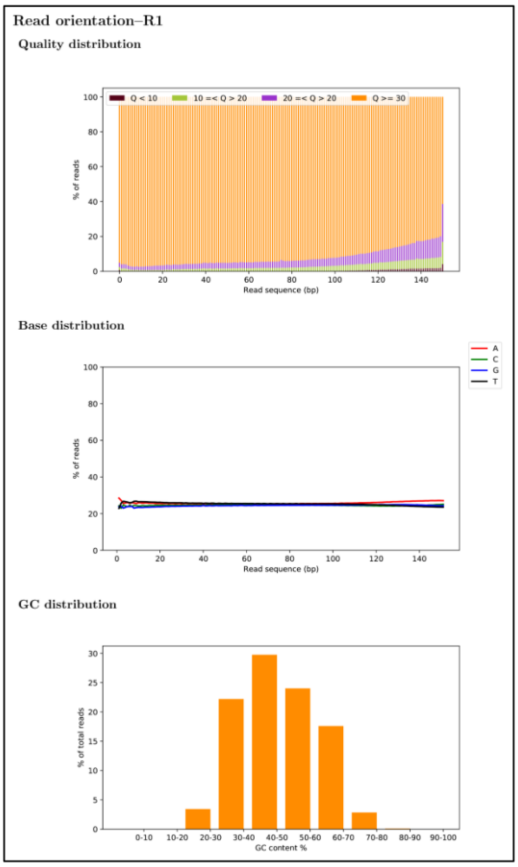

G8

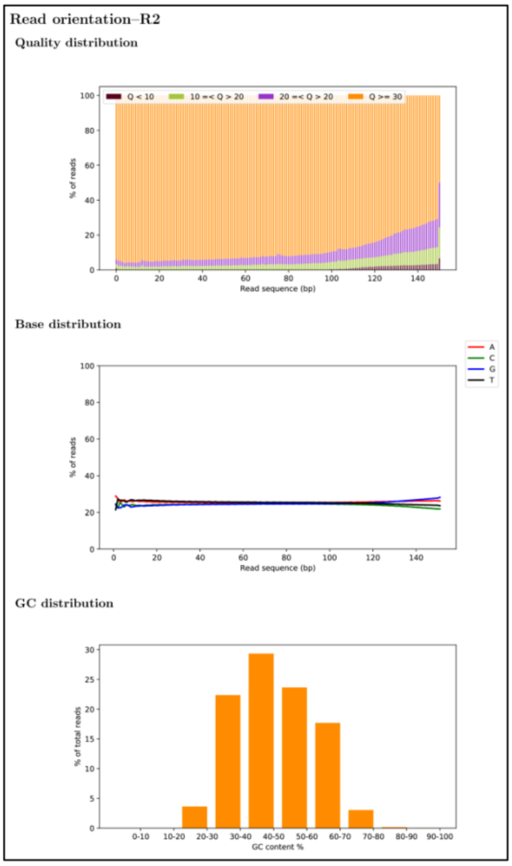

Figure S1. Continued.

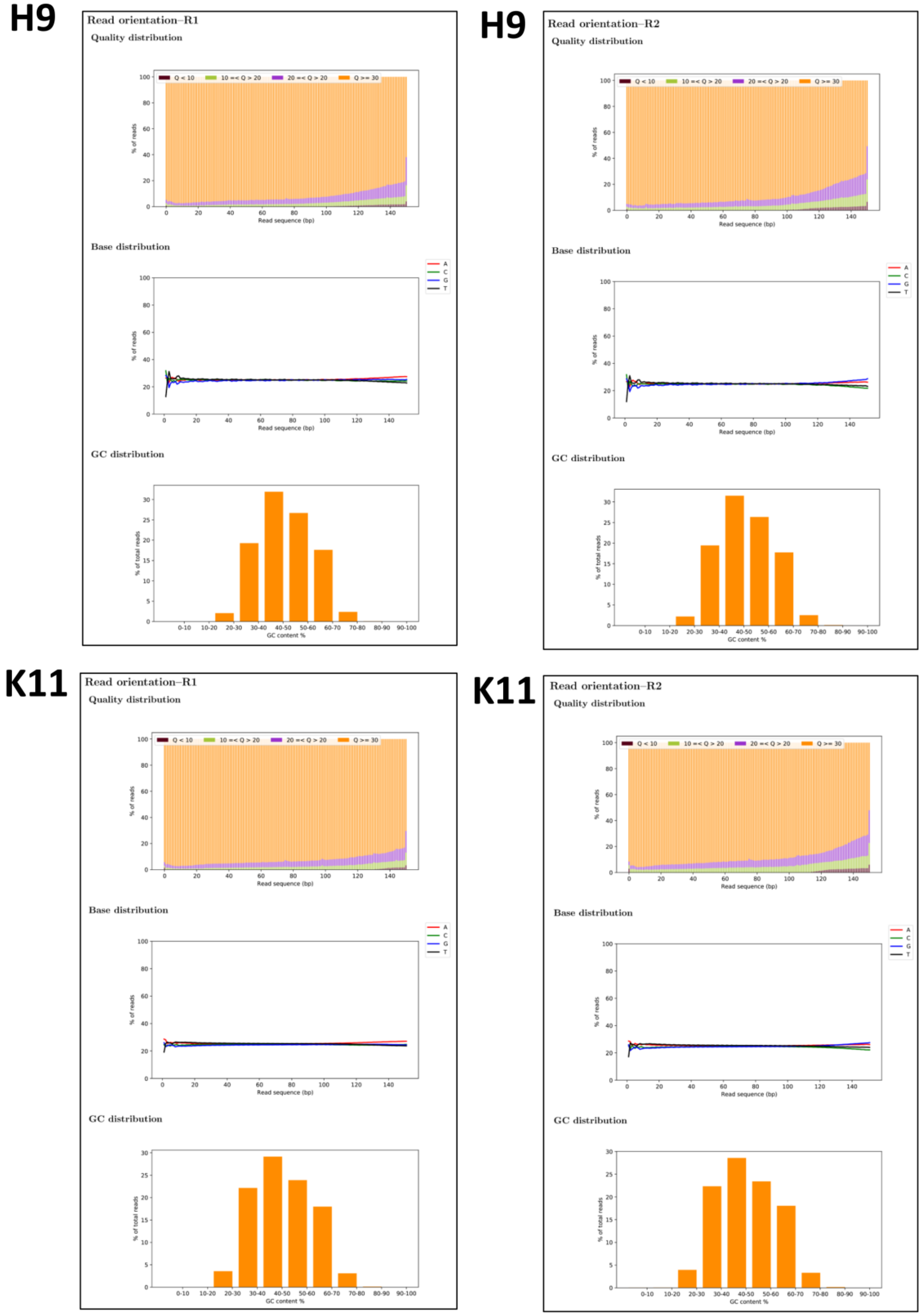

Figure S1. Continued.

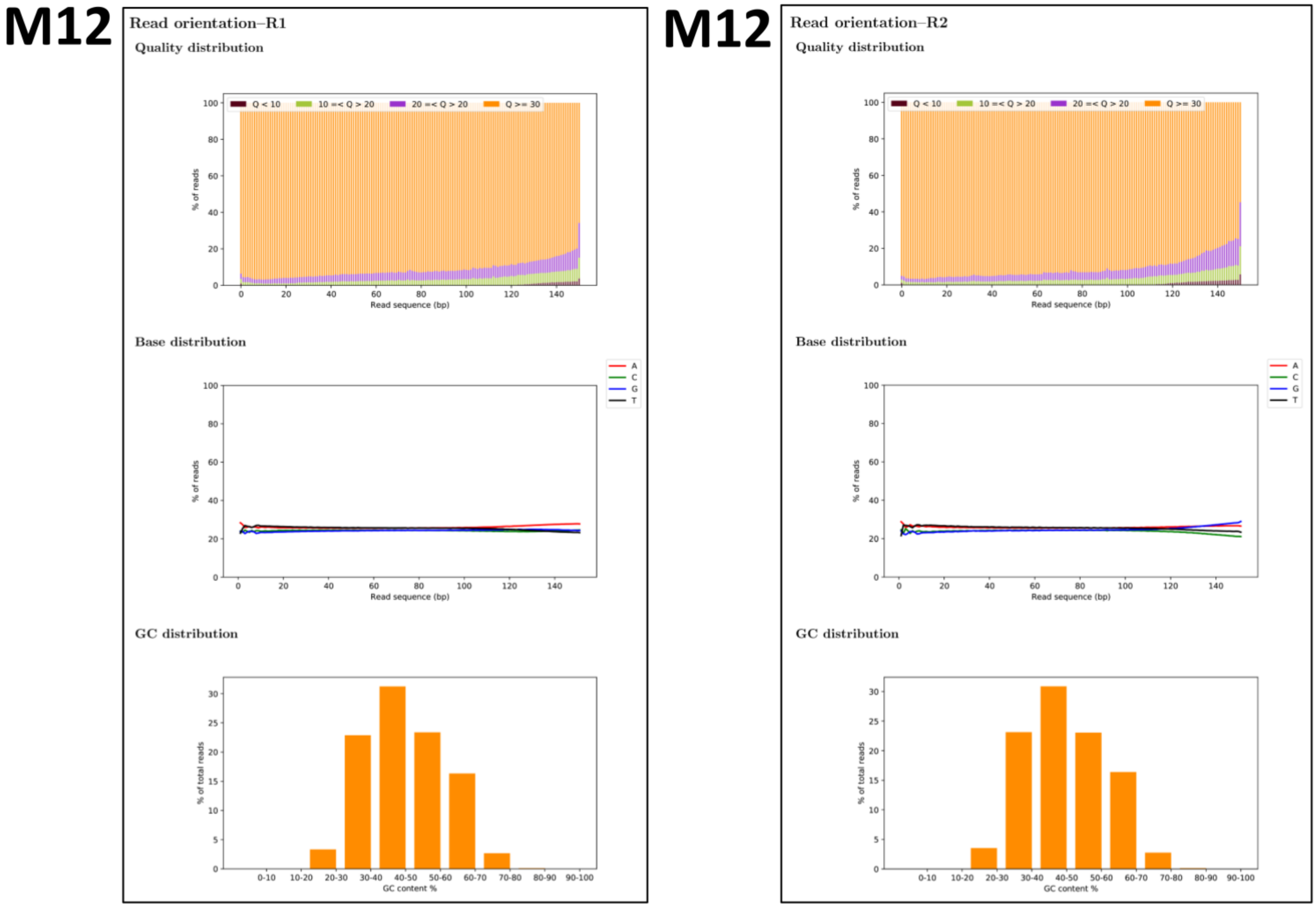

Figure S1. Continued.

Table S2. Alignment Summary

Sample Names	TOTAL READS	TOTAL ALIGNED	TOTAL PASSED ALIGNMENT	TOTAL CROSSMAPPED	TOTAL UNALIGNED
**F7**	72150712	72149198	70728453 (98.03%)	87911 (0.12%)	2740
**H9**	80766664	80593492	78555988(97.26%)	155550(0.19%)	194468
**D5**	70965600	70962916	69452491 (97.87%)	86232 (0.12%)	3962
**C4**	65293460	65290814	63973588(97.98%)	113019(0.17%)	3513
**K11**	70804492	70800392	69253277(97.81%)	168623(0.24%)	5442
**M12**	72730512	72729088	71311719(98.05%)	96650(0.13%)	2884
**G8**	71739982	71738344	70262393 (97.94%)	95059 (0.13%)	2881

Table S3: Coverage Statistics

STATISTICS	C4	D5	G8	K11	M12	H9	F7
PANEL COVERAGE PERCENTAGE	99.2699	99.32332	99.32033	99.33097	99.28246	99.17543	99.23325
PANEL AVG DEPTH	92.71648	93.76819	97.88315	93.54067	99.79161	111.0506	98.19158
READ ONTARGET PERCENTAGE	91.58323	89.68186	90.39168	89.90594	91.3209	91.00202	90.65963

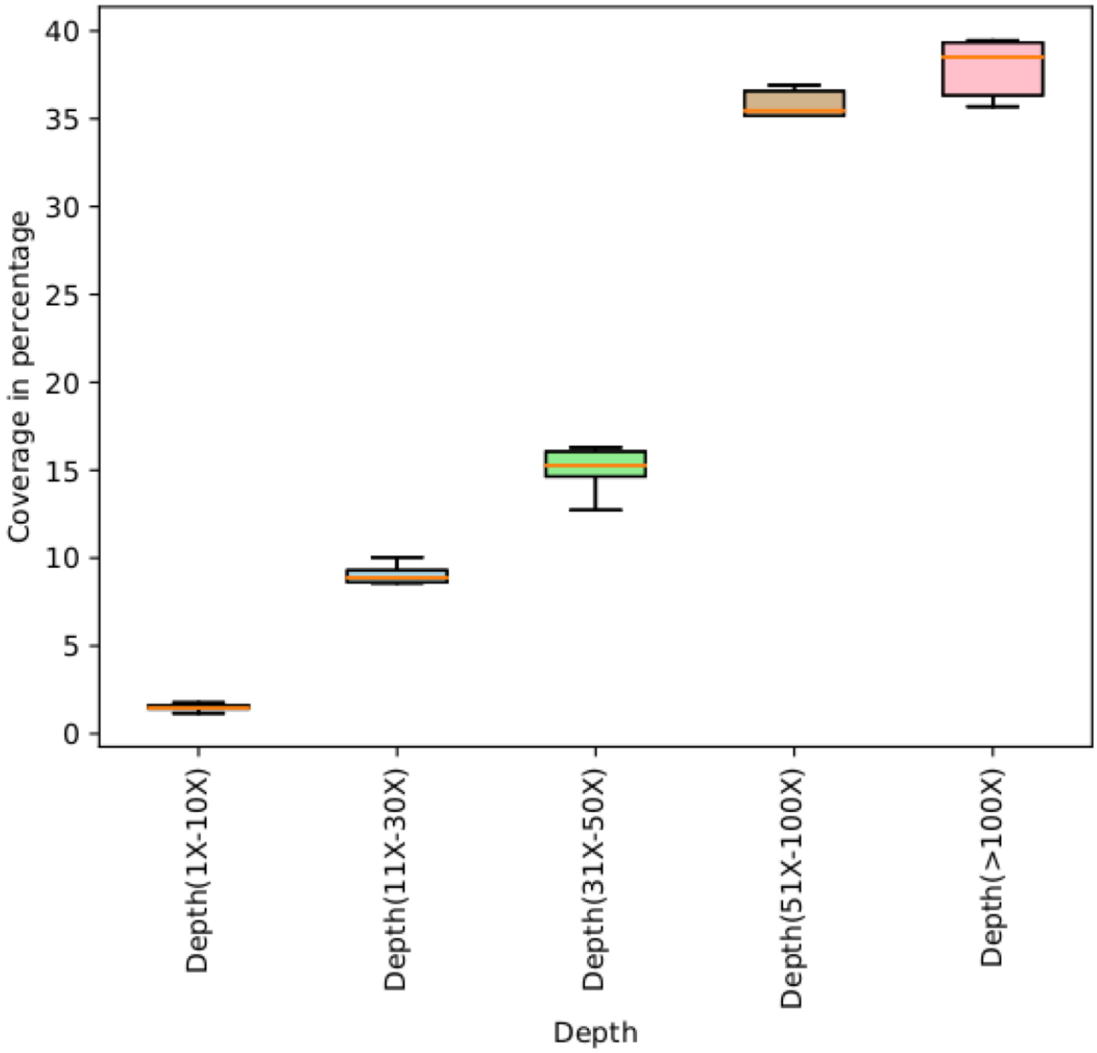

Figure S2. Distribution of Depth. The X-axis represents the Depth in a series of intervals and the Y-axis represents coverage in percentage.

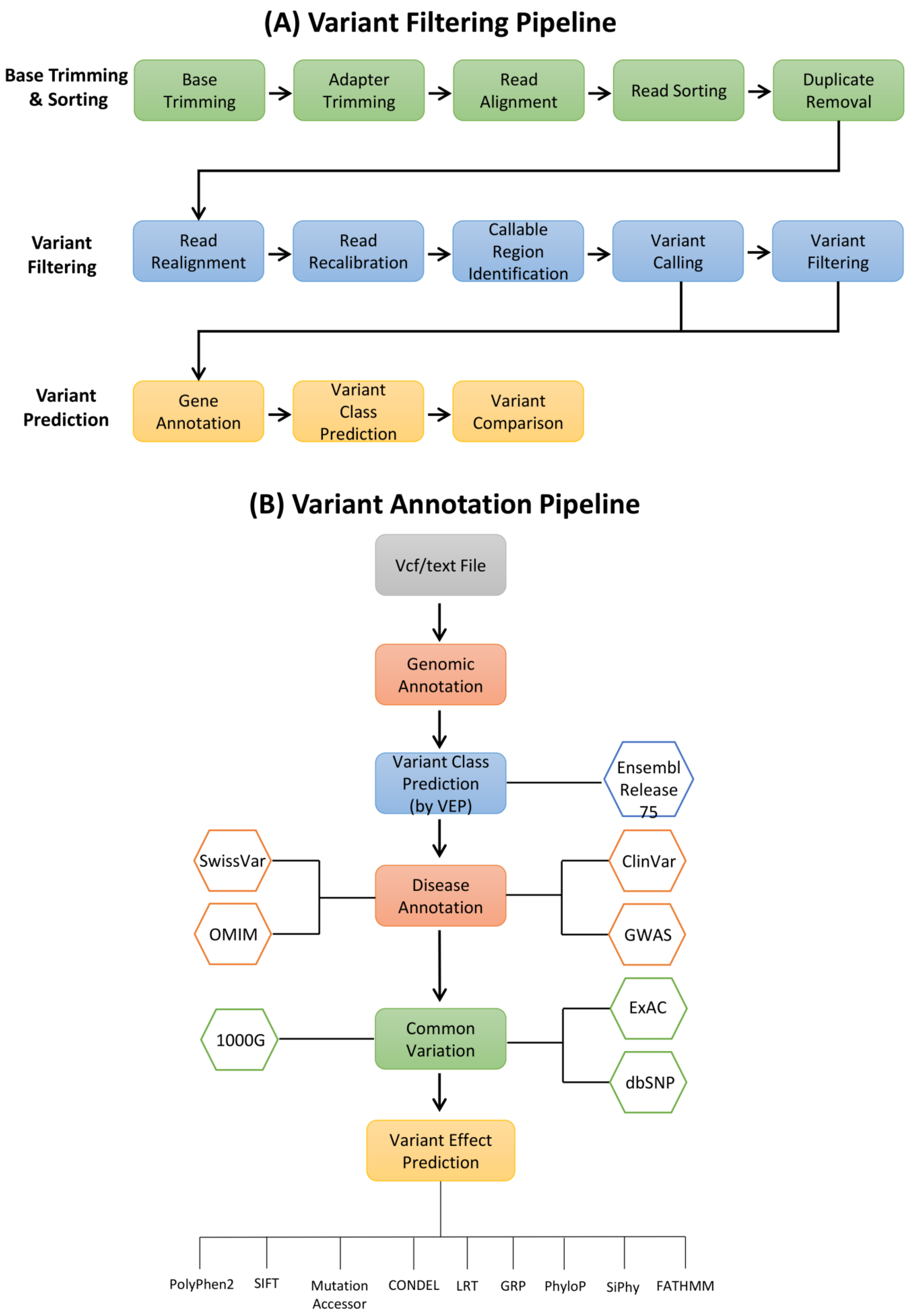

Figure S3. Flowchart depicting the variant filtering and variant annotation pipelines used in the study.

Table S4. Variant Statistics

STATISTICS	K11	F7	M12	G8	D5	H9	C4
TOTAL VARIANTS	72631	72578	71859	72756	72131	71582	70243
TOTAL SNPs	64552	64317	63833	64618	64001	63669	62533
TOTAL INDELs	8079	8261	8026	8138	8130	7913	7710
TOTAL HET	44812	43198	43408	44243	44286	43083	40057
TOTAL HOM	27819	29380	28451	28513	27845	28499	30186
AVERAGE DEPTH	89.3948	91.75552	93.26466	92.67544	88.67584	104.4907	87.85621
MIN READ DEPTH	10	10	10	10	10	10	10
MAX READ DEPTH	4196	4960	3822	4379	4876	4276	3119
AVERAGE VARIANT QUAL	1601.9172	1679.093	1602.515	1644.353	1582.107	1828.829	1585.79
MIN VARIANT QUAL	50.73	50.73	50.73	50.37	50.73	50.73	50.73
MAX VARIANT QUAL	119762	144553	117735	125719	140937	124170	86802.8

Table S5. Variant Class Summary

STATISTICS	H9	K11	C4	M12	D5	G8	F7
Silent	10722	10822	10467	10668	10677	10851	10762
Missense	10846	10906	10608	10785	10815	10850	10909
Nonsense	151	141	150	144	140	141	143
Startloss	55	62	49	59	51	56	49
Stoploss	58	65	61	64	62	65	63
Frameshift- indel	337	356	331	354	340	366	362
Inframe-indel	247	263	230	237	251	256	253

Table S6. Variant Statistics for probable causative positions.

Sample Name	Total Variants	SNPs	Insertions	Deletions	SNP Transitions/Transversions	Total Het/Hom ratio
C4	41576	36227	2467	2882	2.36 (36513/15477)	1.32 (23644/17932)
F7	46377	40316	2721	3340	2.34 (39397/16831)	1.55 (28189/18188)
H9	44701	38998	2603	3100	2.36 (38036/16124)	1.59 (27434/17267)
M12	43099	37595	2507	2997	2.33 (36280/15559)	1.65 (26833/16266)
D5	45472	39640	2686	3146	2.35 (38388/16355)	1.65 (28337/17135)
G8	46067	40111	2742	3214	2.36 (38360/16243)	1.78 (29500/16567)
K11	46828	40764	2732	3332	2.38 (39464/16560)	1.70 (29461/17367)

Table S7. Variant Class Statistics for probable causative positions.

STATISTICS	M12	C4	D5	K11	F7	G8	H9
MISSENSE	2525	2439	2508	2613	2567	2581	2591
SILENT	2661	2595	2675	2781	2728	2773	2720
NONSENSE	22	27	20	26	22	23	24
STARTLOSS	8	6	7	9	8	11	7
STOPLOSS	12	10	12	14	13	11	14
FRAMESHIFT- DEL	37	39	34	44	46	48	42
FRAMESHIFT- INS	45	41	43	40	36	46	36
INFRAME-INS	37	36	37	41	37	46	39
INFRAME-DEL	45	43	43	47	45	48	41

Table S8. Variant Statistics for probable causative Genes.

Sample Name	Total Variants
C4	1394
D5	1709
F7	1746
G8	1700
H9	1749
K11	1887
M12	1589

Table S9. Variant class Statistics for probable causative Genes.

STATISTICS	D5	H9	K11	G8	M12	F7	C4
MISSENSE	67	63	74	79	62	68	62
SILENT	74	71	79	73	73	69	67

Table S10. Summary of observed probable causative variants.

Gene_Name	CHROM_POS	cDNA_Change	AA_Change	Variant_Type	Genotype	snpEff_Impact
ABCA13	chr7:48411769	c.10808T>C	p.Met3603Thr	missense_variant	Het	MODERATE
ABCA2	chr9:139915947	c.884G>A	p.Arg295Gln	missense_variant	Het	MODERATE
ABCC6	chr16:16308285	c.496C>T	p.Arg166Cys	missense_variant	Het	MODERATE
ADAMTS13	chr9:136298777	c.1261C>T	.	structural_interaction_variant	Het	HIGH
ADAMTSL3	chr15:84553914	c.1022A>G	p.His341Arg	missense_variant	Het	MODERATE
ANK3	chr10:61946612	c.1946C>T	p.Thr649Ile	missense_variant	Het	MODERATE
APOB	chr2:21255379	c.1199G>A	p.Arg400His	missense_variant	Het	MODERATE
ARSD	chrX:2833643	c.954G>T	p.Gln318His	missense_variant	Het	MODERATE
ATG16L1	chr2:234173756	c.608A>G	p.Asn203Ser	missense_variant	Het	MODERATE
ATP11A	chr13:113514610	c.2737G>A	p.Asp913Asn	missense_variant	Het	MODERATE
BCL11B	chr14:99641690	c.1483G>A	p.Ala495Thr	missense_variant	Het	MODERATE
CA1	chr8:86249206	c.322C>T	p.His108Tyr	missense_variant	Het	MODERATE
CCAR2	chr8:22476438	c.2431A>G	p.Asn811Asp	missense_variant	Het	MODERATE
CCDC183	chr9:139694856	c.454G>A	p.Glu152Lys	missense_variant	Het	MODERATE
CD101	chr1:117554282	c.535C>T	p.His179Tyr	missense_variant	Het	MODERATE
CD200	chr3:112063809	c.170T>C	p.Val57Ala	missense_variant & splice_region_variant	Het	MODERATE
CDC27	chr17:45214633	c.1816T>G	p.Tyr606Asp	missense_variant	Het	MODERATE
CDC27	chr17:45214669	c.1780A>C	p.Lys594Gln	missense_variant	Het	MODERATE
CDC27	chr17:45214699	c.1750A>G	p.Ser584Gly	missense_variant	Het	MODERATE
CDC27	chr17:45221273	c.1164T>G	p.Phe388Leu	missense_variant	Het	MODERATE
CDC27	chr17:45221286	c.1151G>A	p.Ser384Asn	missense_variant	Het	MODERATE
CDC27	chr17:45234298	c.823C>G	p.Leu275Val	missense_variant	Het	MODERATE
CDC27	chr17:45234343	c.778A>C	p.Asn260His	missense_variant	Het	MODERATE
CDC27	chr17:45249365	c.169G>A	p.Ala57Thr	missense_variant	Het	MODERATE
CDC27	chr17:45249391	c.143A>C	p.Tyr48Ser	missense_variant	Het	MODERATE
CDH23	chr10:73537483	c.4892C>T	p.Ala1631Val	missense_variant	Comp_Het	MODERATE
CDX1	chr5:149546749	c.310C>T	p.Pro104Ser	missense_variant	Het	MODERATE
CGN	chr1:151506533	c.2825G>A	p.Arg942Gln	missense_variant	Het	MODERATE
CHAD	chr17:48545520	c.655C>T	p.Arg219Trp	missense_variant	Het	MODERATE
CHRNA9	chr4:40356515	c.1418T>G	p.Leu473Trp	missense_variant	Het	MODERATE
CNTN2	chr1:205030396	c.821G>A	p.Arg274His	missense_variant	Het	MODERATE
COL11A2	chr6:33143391	c.2336C>T	p.Pro779Leu	missense_variant	Het	MODERATE
COL18A1	chr21:46876414	c.970G>A	p.Gly324Arg	missense_variant	Het	MODERATE
COL26A1	chr7:101192481	c.970C>T	p.Pro324Ser	missense_variant	Het	MODERATE
COL3A1	chr2:189859310	c.1337G>A	p.Arg446His	missense_variant	Het	MODERATE
COL4A3	chr2:228118353	c.764C>T	p.Thr255Met	missense_variant & splice_region_variant	Het	MODERATE
COL5A1	chr9:137582841	c.193C>T	p.Arg65Trp	missense_variant	Het	MODERATE
CPS1	chr2:211521333	c.3661A>G	p.Ile1221Val	missense_variant	Het	MODERATE
CTAGE4	chr7:143964267	c.2077T>C	p.Phe693Leu	missense_variant	Het	MODERATE
CTBP2	chr10:126683123	c.2315T>G	p.Leu772Trp	missense_variant	Het	MODERATE
CTBP2	chr10:126683162	c.2276G>A	p.Arg759Gln	missense_variant	Het	MODERATE
CYC1	chr8:145150013	c.11C>T	p.Ala4Val	missense_variant	Het	MODERATE
CYP4A11	chr1:47407062	c.44T>C	p.Val15Ala	missense_variant	Het	MODERATE
DDX23	chr12:49239376	c.190C>T	p.Arg64Cys	missense_variant	Het	MODERATE
DGKD	chr2:234345020	c.752T>G	p.Val251Gly	missense_variant	Het	MODERATE
DLGAP2	chr8:1497493	c.634G>A	p.Gly212Ser	missense_variant	Het	MODERATE
DNA2	chr10:70192040	c.1796G>A	p.Arg599His	missense_variant	Het	MODERATE
DNAH11	chr7:21840783	c.10055C>T	p.Thr3352Met	missense_variant	Het	MODERATE
DNAH6	chr2:84934697	c.8905G>T	p.Ala2969Ser	missense_variant	Het	MODERATE
DYSF	chr2:71816729	c.3409G>A	p.Val1137Met	missense_variant	Het	MODERATE
ECM1	chr1:150484263	c.1120C>T	p.Arg374Cys	missense_variant	Het	MODERATE

ELP2	chr18:33747114	n.2015-2A>G	.	splice_acceptor_variant & intron_variant	Het	HIGH
ENAH	chr1:225706951	c.751T>G	p.Leu251Val	missense_variant	Het	MODERATE
EPPK1	chr8:144941229	c.6193T>A	p.Tyr2065Asn	missense_variant	Het	MODERATE
EZR	chr6:159197483	c.752A>G	p.Asn251Ser	missense_variant	Het	MODERATE
FAT4	chr4:126371616	c.9451G>A	p.Ala3151Thr	missense_variant	Hom	MODERATE
FOLH1	chr11:49207245	c.802C>T	p.Leu268Phe	missense_variant	Het	MODERATE
FSIP2	chr2:186657220	c.5357A>G	p.Asn1786Ser	missense_variant	Het	MODERATE
GBX1	chr7:150845855	c.913G>A	p.Val305Ile	missense_variant	Het	MODERATE
GJB2	chr13:20763228	c.493C>T	p.Arg165Trp	missense_variant	Het	MODERATE
GPR45	chr2:105858875	c.560C>T	p.Thr187Met	missense_variant	Het	MODERATE
HK3	chr5:176317824	c.533G>C	p.Arg178Thr	missense_variant & splice_region_variant	Het	MODERATE
HMCN1	chr1:186157125	c.16525C>T	p.Arg5509Trp	missense_variant	Het	MODERATE
HNRNPDL	chr4:83350731	c.113T>C	p.Leu38Pro	missense_variant	Het	MODERATE
HSD3B2	chr1:119964578	c.454A>G	p.Thr152Ala	missense_variant	Het	MODERATE
HSD3B2	chr1:119964590	c.466T>C	p.Tyr156His	missense_variant	Het	MODERATE
IFT140	chr16:1630800	c.1484A>G	p.Tyr495Cys	missense_variant	Het	MODERATE
IGSF3	chr1:117142868	c.1784G>A	p.Trp595*	stop_gained	Het	HIGH
IGSF3	chr1:117156459	c.760G>A	p.Asp254Asn	missense_variant	Het	MODERATE
KCNH6	chr17:61615462	c.1538C>T	p.Ala513Val	missense_variant	Het	MODERATE
KCNQ4	chr1:41289870	c.1232G>A	p.Arg411His	missense_variant	Het	MODERATE
KIF13B	chr8:29037656	c.685A>G	.	structural_interaction _variant	Het	HIGH
KMT2B	chr19:36212437	c.2188G>A	p.Gly730Arg	missense_variant	Het	MODERATE
KMT5A	chr12:123892186	c.995T>C	p.Leu332Pro	missense_variant	Het	MODERATE
KRT77	chr12:53086287	c.1345T>C	p.Tyr449His	missense_variant	Het	MODERATE
LAMA4	chr6:112438959	c.4964G>A	p.Gly1655Glu	missense_variant	Het	MODERATE
LAMA5	chr20:60885845	c.10322C>T	p.Thr3441Met	missense_variant	Het	MODERATE
LMAN2	chr5:176765576	c.346G>A	p.Val116Ile	missense_variant	Het	MODERATE
LRP1	chr12:57589660	c.8575G>A	p.Gly2859Ser	missense_variant	Het	MODERATE
MET	chr7:116339851	c.713T>C	.	structural_interaction _variant	Het	HIGH
MYO15A	chr17:18046138	c.5894G>A	p.Arg1965His	missense_variant	Het	MODERATE
MYO3B	chr2:171248911	c.1697G>A	p.Gly566Glu	missense_variant	Het	MODERATE
NFE2L3	chr7:26224939	c.1621C>T	p.Arg541Cys	missense_variant	Het	MODERATE
NPC2	chr14:74953134	c.88G>A	p.Val30Met	missense_variant	Het	MODERATE
NPHP4	chr1:5935048	c.2930C>T	p.Thr977Met	missense_variant	Het	MODERATE
NPHP4	chr1:5964791	c.2029C>T	p.Pro677Ser	missense_variant	Het	MODERATE
NUDT14	chr14:105643046	c.253G>A	p.Gly85Arg	missense_variant	Het	MODERATE
OTOF*ƚ	**chr2:26702224**	**c.2122C>T**	**p.Arg708***	**stop_gained**	**Hom**	**HIGH**
OTOG	**chr11:17632285**	**c.5438T>G**	**p.Val1813Gly**	**missense_variant**	**Het**	**MODERATE**
PADI2	chr1:17396693	c.1654C>T	p.Arg552Cys	missense_variant	Het	MODERATE
PCDH19	chrX:99551307	c.3415G>A	p.Gly1139Ser	missense_variant	Hom	MODERATE
PCDHGA8	chr5:140772831	c.451C>G	p.Arg151Gly	missense_variant	Het	MODERATE
PCNT	chr21:47860930	c.9556C>T	p.Arg3186Trp	missense_variant	Het	MODERATE
PDE6C	chr10:95422392	c.2359G>C	p.Val787Leu	missense_variant	Het	MODERATE
PDIA6	chr2:10932003	c.658G>A	p.Asp220Asn	missense_variant	Het	MODERATE
PIEZO1	chr16:88804838	c.645C>G	p.His215Gln	missense_variant	Het	MODERATE
PLG	chr6:161152909	c.1571C>T	p.Ala524Val	missense_variant	Het	MODERATE
POLE	chr12:133208960	c.6271C>T	p.Pro2091Ser	missense_variant	Het	MODERATE
POPDC3	chr6:105609321	c.464T>A	p.Leu155His	missense_variant	Het	MODERATE
PREX2	chr8:69028025	c.3184C>T	p.Arg1062Cys	missense_variant	Het	MODERATE
PTCH1	chr9:98209306	c.4232A>G	p.Asp1411Gly	missense_variant	Het	MODERATE
PYGL	chr14:51404515	c.284C>T	p.Thr95Ile	missense_variant	Het	MODERATE
RAB13	chr1:153955263	c.346C>T	p.Arg116Cys	missense_variant	Het	MODERATE
RDH12	chr14:68191267	c.146C>T	p.Thr49Met	missense_variant	Het	MODERATE

RHPN1	chr8:144461673	c.940G>A	p.Ala314Thr	missense_variant	Het	MODERATE
RYR3	chr15:33952507	c.4505G>T	p.Arg1502Leu	missense_variant	Het	MODERATE
SBF2	chr11:10011136	c.1303G>A	p.Ala435Thr	missense_variant	Het	MODERATE
SHANK1	chr19:51171270	c.3947G>A	p.Gly1316Asp	missense_variant	Het	MODERATE
SHC3	chr9:91628436	c.1711C>G	p.Leu571Val	missense_variant	Het	MODERATE
SLC26A4**	**chr7:107340581**	**c.1668T>A**	**p.Tyr556***	**stop_gained**	**Het**	**HIGH**
SLC5A8	chr12:101588886	c.524T>G	p.Phe175Cys	missense_variant	Het	MODERATE
SLC6A9	chr1:44489938	c.12A>C	p.Lys4Asn	missense_variant	Het	MODERATE
SMARCA2	chr9:2039627	c.517C>T	p.Pro173Ser	missense_variant	Het	MODERATE
SNTG2	chr2:1079293	c.162T>G	p.Ile54Met	missense_variant	Het	MODERATE
SPC25	chr2:169733775	c.319A>C	p.Lys107Gln	missense_variant	Het	MODERATE
SPEF2	chr5:35793307	c.4601A>C	p.Asp1534Ala	missense_variant	Het	MODERATE
SPTBN5	chr15:42169085	c.3773G>T	p.Ser1258Ile	missense_variant	Het	MODERATE
SRCAP	chr16:30740333	c.5705A>G	p.Glu1902Gly	missense_variant	Het	MODERATE
SYT8	chr11:1857809	c.716C>T	p.Pro239Leu	missense_variant	Het	MODERATE
TBL3	chr16:2026871	c.1349C>T	p.Pro450Leu	missense_variant	Het	MODERATE
TCOF1	chr5:149767544	c.2939C>T	p.Ala980Val	missense_variant	Het	MODERATE
THAP4	chr2:242545814	c.79G>T	.	structural_interaction _variant	Het	HIGH
TOP3B	chr22:22314764	c.1583C>T	p.Thr528Met	missense_variant	Het	MODERATE
TRPM5	chr11:2434057	c.2282C>T	p.Pro761Leu	missense_variant	Het	MODERATE
UNC45A	chr15:91492564	c.1897C>T	p.Arg633Trp	missense_variant	Het	MODERATE
WFS1	chr4:6303902	c.2380G>A	p.Glu794Lys	missense_variant	Het	MODERATE
WIPI1	chr17:66453538	c.25C>T	p.Pro9Ser	missense_variant	Het	MODERATE
XRCC1	chr19:44058892	c.320G>A	p.Arg107His	missense_variant	Het	MODERATE

*****Very rare variant (no homozygous variant was found in ExAC, 1000G and GenomAD databases).

ƚ Pathogenic mutation in the present study.

**Novel gene mutation (not found in ExAC, 1000G and GenomAD databases).

ClinVar submissions from the current study: *OTOF* (SCV001448680.1), *OTOG* (SCV001448682.1) and *SLC26A4* (SCV001448681.1).

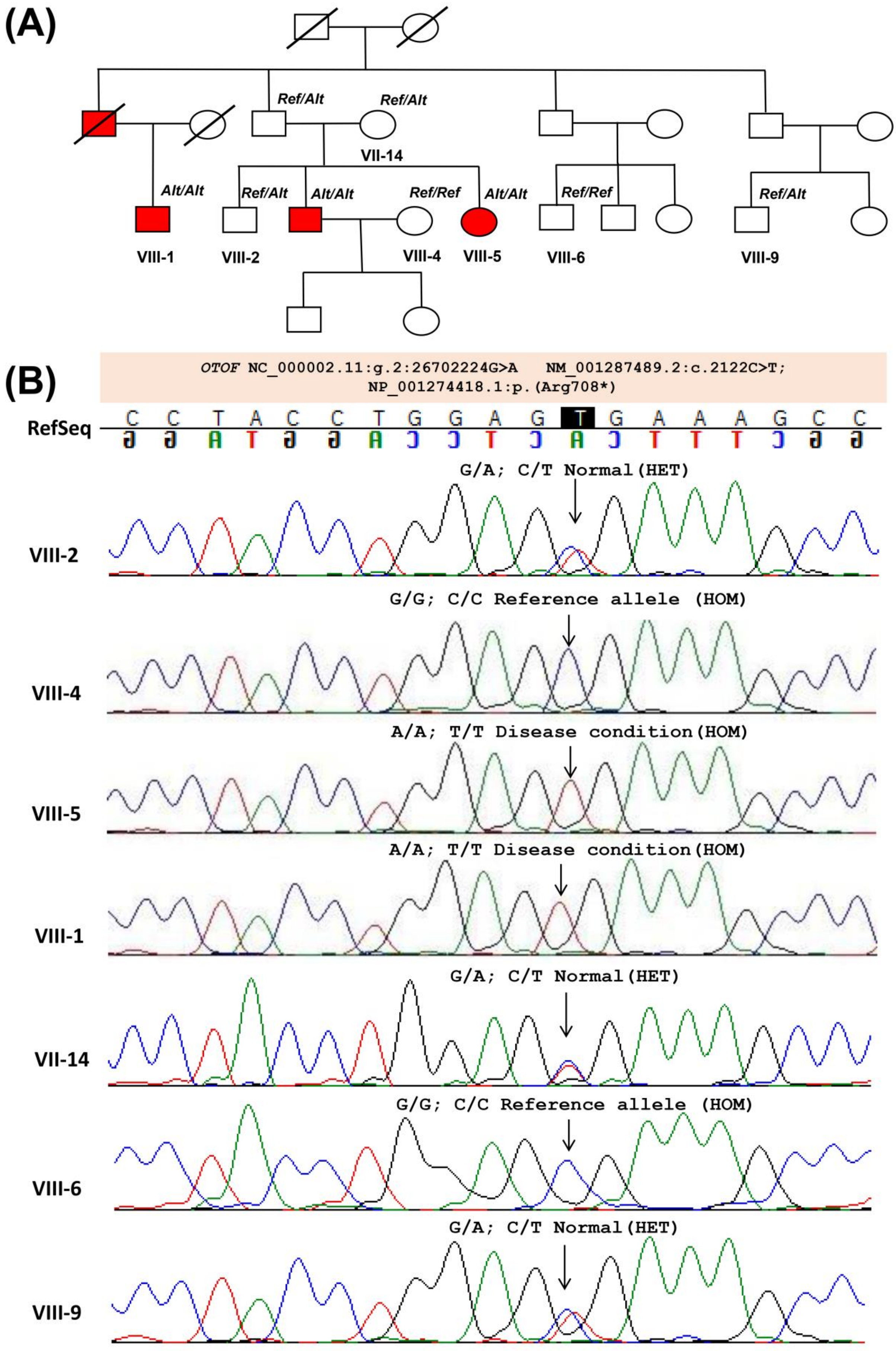

Figure S4. Variant segregation and Sanger sequencing. (A) Pedigree showing the inheritance and *OTOF* variant segregation in seven samples (B) Sanger sequencing data to reconfirm the mutation identified by WES.

NC_000002.12:g.2:26479356G>A

NM_194248.2:c.2122C>T

NP_001274418.1:p.(Arg708*)

rs80356590

(A)

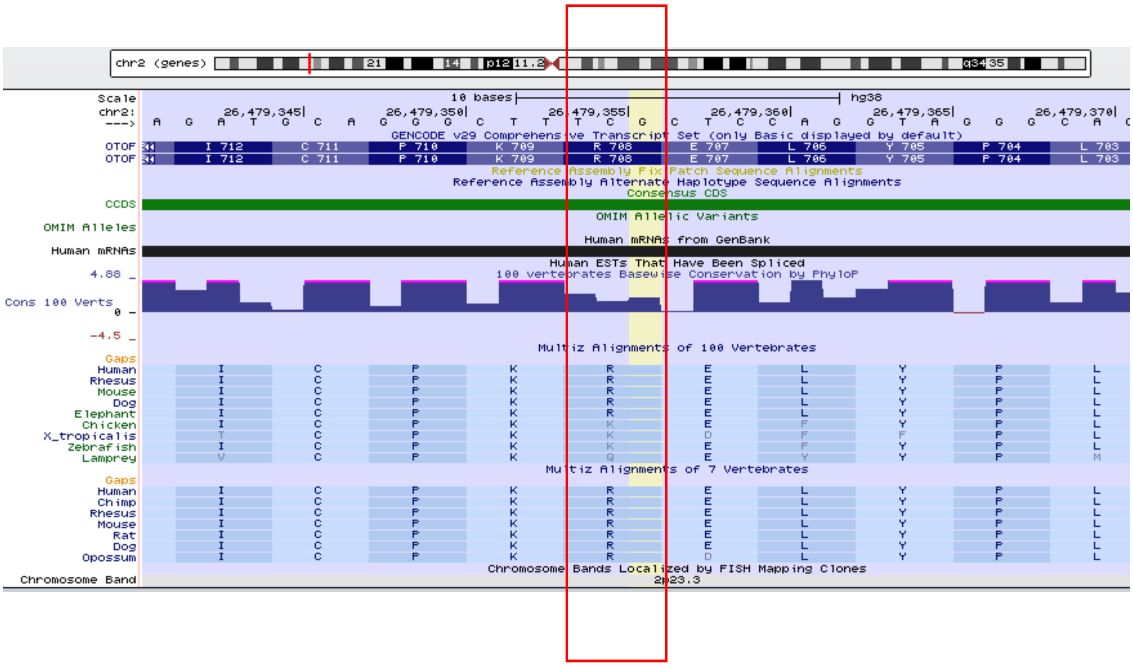

(B)

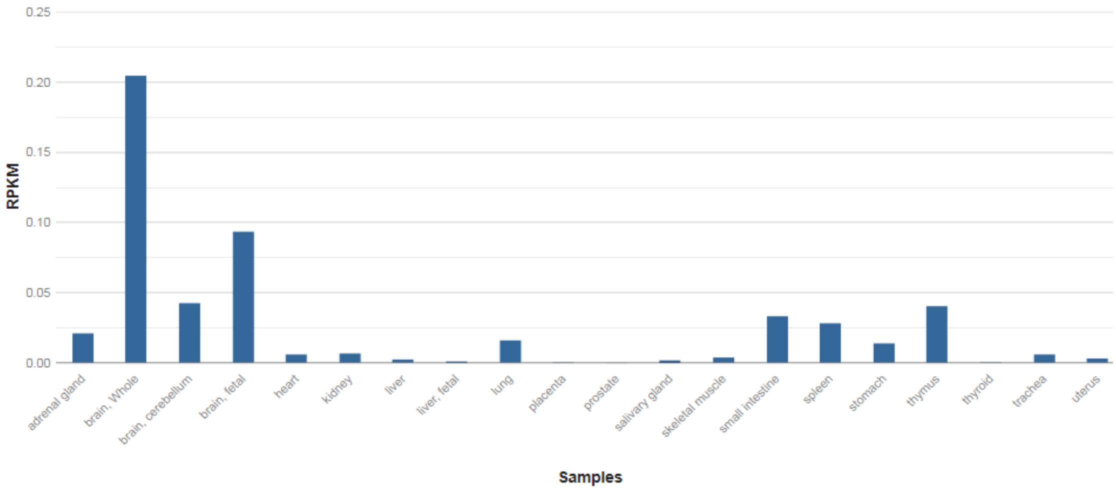

Figure S5. (A) Screenshot from UCSC Genome Browser showing the conserved region of the *OTOF* gene via Multiz alignments of 7 vertebrates (B) *OTOF* gene expression (mRNA) from 20 human tissues (Source: NCBI, Gene ID: 9381 BioProject: PRJNA280600 updated: 26-06-2019).
